# Quantifying ADHD Symptoms in Open-Ended Everyday Life Contexts With a New Virtual Reality Task

**DOI:** 10.1177/10870547211044214

**Published:** 2021-12-05

**Authors:** Erik Seesjärvi, Jasmin Puhakka, Eeva T. Aronen, Jari Lipsanen, Minna Mannerkoski, Alexandra Hering, Sascha Zuber, Matthias Kliegel, Matti Laine, Juha Salmi

**Affiliations:** 1 University of Helsinki, Helsinki, Finland; 2 Child Neurology, University of Helsinki and Helsinki University Hospital, Finland; 3 Child Psychiatry, University of Helsinki and Helsinki University Hospital, Finland; 4 Tilburg University, Tilburg, The Netherlands; 5 University of Geneva, Switzerland; 6 Swiss National Centre of Competence in Research LIVES, Switzerland; 7 Åbo Akademi University, Turku, Finland; 8 University of Turku, Turku, Finland; 9 Department of Neuroscience and Biomedical Engineering, Aalto University, Espoo, Finland

**Keywords:** ADHD, executive function, naturalistic behavior, real-world attention, virtual reality

## Abstract

**Objective:**

To quantify goal-directed behavior and ADHD symptoms in naturalistic conditions, we developed a virtual reality task, EPELI (Executive Performance in Everyday LIving), and tested its predictive, discriminant and concurrent validity.

**Method:**

We collected EPELI data, conventional neuropsychological task data, and parent-ratings of executive problems and symptoms in 38 ADHD children and 38 typically developing controls.

**Results:**

EPELI showed predictive validity as the ADHD group exhibited higher percentage of irrelevant actions reflecting lower attentional-executive efficacy and more controller movements and total game actions, both indicative of hyperactivity-impulsivity. Further, the five combined EPELI measures showed excellent discriminant validity (area under curve 88 %), while the correlations of the EPELI efficacy measure with parent-rated executive problems (*r* = .57) and ADHD symptoms (*r* = .55) pointed to its concurrent validity.

**Conclusion:**

We provide a proof-of-concept validation for a new virtual reality tool for ecologically valid assessment of ADHD symptoms.

## Introduction

Attention deficit hyperactivity disorder (ADHD), characterized by the symptoms of inattention, hyperactivity, and impulsivity, is among the most common neurodevelopmental disorders, with an estimated 5.9% world-wide prevalence at childhood (Faraone et al., 2021). ADHD diagnostics still rely largely on interviews and questionnaires prone to reporter’s subjective bias, while the research on the predictive value and clinical utility of the current objective experimental test methods (Hall et al., 2016; Nichols & Waschbusch, 2004) and potential biomarkers (Mehta et al., 2020) is underway. To improve diagnostics and increase our understanding on ADHD, it would be important to establish methods that can characterize attentional-executive deficits in ADHD both objectively and accurately (Berger & Goldzweig, 2010; Gualtieri & Johnson, 2005). For this purpose, we developed a new virtual reality (VR) game that taps attention and executive function in complex life-like situations, and administered it to children with versus without ADHD.

ADHD is associated with several adverse outcomes such as impairments in quality of life, emotional and social impairments, and educational underachievement (Faraone et al., 2021), as well as with impairments in multiple cognitive domains as measured by conventional task paradigms (Pievsky & McGrath, 2018). However, how well the existing task paradigms capture the cognitive phenomena related to observed outcomes remains controversial. An important caveat in the conventional experimental methods in ADHD assessment relates to their highly structured nature and the assumption that maximal performance in a simple task is an informative predictor of how ADHD symptoms manifest in the complex and varied situations that characterize everyday life (Barkley & Murphy, 2010; Parsons et al., 2017). However, monotonous task structures where the participants are forced to constantly work at their capacity limits do not represent typical goal-directed behavior in everyday situations, where the goals are composed of dynamically changing cascades of daily actions (Ackerman, 1994; Toplak et al., 2013). In the rich and meaningful everyday environments, there are also large amounts of contextual information that trigger and support goal-directed behaviors (e.g., Marsh et al., 2008), which is lacking from simplified tasks with restricted stimulus sets. Moreover, such tasks may not capture the inter-individual variability in various types of maladaptive behaviors in daily attentional-executive functions that the diagnostic systems are targeting. Hence, it is not surprising that measures and behavioral observations in contextually simple tasks have limited predictive validity to the complex real-life settings where children with ADHD live and where their symptoms emerge (Barkley & Murphy, 2010, 2011; Hall et al., 2016). Furthermore, accumulating brain imaging findings suggest that ADHD is not captured by “capacity-based” descriptions but rather manifests itself as a condition where the usage of cognitive resources fluctuates excessively in time (Sonuga-Barke & Castellanos, 2007). Taken together, there is a need for measures that can detect difficulties in typical everyday goal-directed behaviors that arise in rich, open-ended, and dynamic environments (e.g., Kingstone et al., 2008). Recent advances in virtual reality (VR) technology and related head-mounted displays (HMD) have provided opportunities for developing such environments (Bohil et al., 2011; Pan & Hamilton, 2018) without losing the accuracy of computerized measurements.

Thus far, the most widely used VR paradigm in ADHD research has been the continuous performance task (CPT), where the participant responds to relevant objects and avoid responding to other pre-designated objects in an ongoing stimulus stream. The application of CPT has been a logical step, as it has been the most consistent cognitive test method in differentiating children with versus without ADHD (see e.g., Albrecht et al., 2015; Ogundele et al., 2011), and its VR versions can provide additional valuable data such as information about body movements (Parsons et al., 2019). Despite these advances, for instance, Parsons et al. (2019) note in their meta-analysis on virtual classroom CPT, that “It is unlikely that the virtual classroom as is currently designed has changed that relationship between computerized testing and self or observer report of real-world executive control difficulties exhibited by those with ADHD” (p. 351). Thus, the full potential of VR to capture naturalistic symptom-related behaviors has not yet been realized (Parsons et al., 2017; see also Ryu et al., 2020).

As a response to the demand for naturalistic and engaging VR tasks that would reflect everyday behaviors, we developed a game coined as EPELI (Executive Performance in Everyday Living, link to a video) and used it to study how ADHD children perform daily chores in an environment akin to those where their symptoms occur. EPELI is inspired by studies examining real-world executive functions in prefrontal patients (e.g., Shallice & Burgess, 1991; see also Rand et al., 2009, for a computerized version using traditional 2D monitor and video capture technology) and contains several scenarios in which the participants perform routine everyday tasks containing multiple elements. To our knowledge, this is the first time when immersive HMD-based VR technology has been used to implement an environment where children need to carry out varied everyday tasks while planning their movement around virtual surroundings, monitoring the time, and avoiding getting distracted by irrelevant objects or events. Each task scenario consists of a spoken list of to-be-done subtasks given prior to executing the scenario. This prospective memory context employed in EPELI carries a strong executive component (e.g., Zuber et al., 2019) by orienting the participants toward shared goals but leaves open the exact way how the required subtasks are planned and executed. At the same time, volitional actions and maladaptive behaviors alike can be accurately quantified. Giving the participant the freedom to interact with an engaging open-ended realistic environment creates an immersive illusion of real life (Bohil et al., 2011; Slater, 2018) that is expected to prompt typical ADHD-related behavior such as impulsive actions toward attractive task-irrelevant stimuli.

By using the rich data EPELI provides, we operationalized measures that reflect the key features of ADHD symptomatology, namely attentional-executive function problems and hyperactivity-impulsivity. Our primary aim was to examine whether participants’ performance and behaviors while playing EPELI show (i) predictive validity by differentiating between ADHD children and typically developing controls, (ii) discriminant validity by differentiating individual children based on their group status (ADHD vs. controls), and (iii) concurrent validity by being associated with parent-rated ADHD symptoms and executive function deficits.

In the preregistration of the study (AsPredicted.org #31918), we set more specific hypotheses that pertain to the three objectives listed above. Concerning predictive and discriminant validity (points (i) and (ii)), we hypothesized that ADHD participants would exhibit a lower percentage of relevant actions out of all actions than typically developing (TD) controls. We also expected that movement trajectories when navigating in the apartment would be longer in ADHD than in TD due to poorer planning and execution skills, and that the sensors attached to the HMD and the controller would detect higher levels of motion in ADHD participants (head and controller hand movements), indicating hyperactivity. ADHD participants were also expected to show more actions overall, reflecting impulsivity. Based on prior VR studies, these group differences were expected to be particularly pronounced in scenarios with more distracting stimuli (Neguţ et al., 2017; Parsons et al., 2007). Moreover, we hypothesized that ADHD participants would show higher variability in the EPELI measures (Sonuga-Barke & Castellanos, 2007) and their performance would not improve during the sequence of EPELI scenarios like in TD controls. As regards concurrent validity (point (iii)), we hypothesized that the EPELI measures, simulating real-life situations, would correlate significantly with ADHD rating measures and questionnaires that screen everyday cognitive abilities. As a secondary issue, we expected that those neuropsychological task performances that yield significant group differences between children with ADHD and neurotypical control children would also correlate with the EPELI measures.

## Materials and Methods

### Participants

In total, 47 children with ADHD and 68 TD controls participated in this study. For children with ADHD, the inclusion criteria were (a) ADHD diagnosis with predominantly hyperactive/impulsive or combined inattention and hyperactive/impulsive subtype (F90) set by a licensed physician following the ICD-10 criteria (World Health Organization, 2016), (b) age of 9 to 12 years when recruited, and (c) native language Finnish. The exclusion criteria were (a) any diseases of the nervous system (ICD-10, G00–G99) and (b) any mental and behavioral disorders (F00–F99) except F93 (Emotional disorder with onset specific to childhood) and F98 (Unspecified behavioral and emotional disorder), which were permitted as secondary diagnoses because of being common comorbidities. For the TD children, the criteria were the same, except that the exclusion criteria included any mental or behavioral disorders (F00–F99). Five children with ADHD and 17 controls were excluded from the final sample due to technical failures or human errors (scenarios accidentally presented in different order). Furthermore, two participants with inattentive subtype of ADHD, one participant with specific developmental disorder of motor function (F82), and one participant with a mixed disorder of scholastic skills (F81.3) were excluded from the ADHD group for not meeting the abovementioned criteria. In the ADHD group, two children had concurrent F93.89 diagnosis (Emotional disorder with onset specific to childhood, difficulties with regulation of emotions) and one child had concurrent F98.9 diagnosis (Unspecified behavioral and emotional disorder). Propensity matching using age, gender, parental education, and familial income as the matching variables was conducted to select the same number of TD participants from the remaining 51 participants. Using R package MatchIt (Ho et al., 2011), both greedy nearest neighbor method and optimal matching method were tried and yielded the same selection of control participants. Thus, the final sample consisted of 38 ADHD and 38 control participants with no group differences in the background variables (see Table 2).

**Table 1.** Correlation Matrix for the Main EPELI Variables (*n* = 76).

	Total score	Task efficacy	Navigation efficacy	Controller motion	Actions
Total score	1				
Task efficacy	.464***	1			
Navigation efficacy	.737***	.812***	1		
Controller motion	−.403***	−.667***	−.658***	1	
Total actions	−.378***	−.836***	−.755***	.762***	1

*Note.* FDR correction.

* *p* ≤ .05. **p ≤ .01. ***p ≤ .001.

**Table 2.** Background Characteristics and Main Questionnaire Sum Scores for the ADHD and TD Groups.

Variable	ADHD group		TD group		Test statistic	*p*
	Mean	*SD*	Mean	*SD*		
Age	10 year 4 month	1 year 1 month	10 year 9 month	1 year 1 month	*t*(74) = −1.70	.093
Handedness	32/5/1		36/1/1		Fisher’s Exact test	.200
Gender	33/5		30/8		Fisher’s Exact test	.544
Parental income^a^	3.7	1.0	4.0	1.0	*t*(74) = −1.15	.253
Parental education^b^	2.4	0.6	2.7	0.5	*t*(74) = −1.88	.063
WISC-IV similarities	23.7	4.4	24.7	5.1	*t*(74) = −0.88	.377
WISC-IV matrix reasoning	19.7	4.0	20.4	5.1	*t*(74) = −0.67	.505
ADHD-RS	31.5	9.4	7.9	6.2	*t*(74) = 12.94	<.001
BRIEF	159.7	20.2	103.5	16.0	*t*(74) = 13.46	<.001
CBCL internalizing problems	9.7	5.7	4.3	3.5	*t*(74) = 4.71	<.001
CBCL externalizing problems	17.6	8.3	4.4	3.9	*t*(74) = 8.87	<.001
EQELI	41.3	17.0	12.8	11.3	*t*(74) = 8.61	<.001
Gaming experience^c^	0.1	1.9	−0.1	2.2	*t*(74) = 0.37	.706
Familiarity of the tasks	5.1	1.3	4.9	0.9	*t*(74) = 0.72	.469
Object naming task	19.5	1.0	19.6	1.1	*t*(74) = −0.45	.654
Simulator sickness	0.6	1.0	0.9	1.0	*t*(74) = −1.11	.269
Presence questionnaire	61.9	11.3	64.9	8.5	*t*(74) = −1.33	.187

a Before tax per adult; 1 = less than 1,500 €/m, 2 = 1,500–2,200 €/m, 3 = 2,200–3,000 €/m, 4 = 3,000–4,000 €/m, 5 = over 4,000 €/m.

b 1 = Comprehensive school, 2 = High school/Vocational school, 3 = University degree or equivalent.

c Sum of normalized scores of three questions (1) “How many days per week you play computer, console or cell phone games?”, (2) “How long is you average playing session”, (3) “How many years have you played regularly?”

The participants with ADHD were recruited at the Helsinki University Hospital by advertising the study at a Child Psychiatric Unit with handouts and phone calls, and by advertising the study through Finnish ADHD Foundation contact channels, at the Espoo City Child Psychiatric Unit, the Vantaa Family Counselling Unit, and a private clinic in Espoo (ProNeuron LTD). The eligibility of each child to participate in the study was initially checked on the first contact (phone call or email) with the parent. For the ADHD group, all diagnoses were controlled for by checking medical documents (e.g., a copy of medical records summary) during the measurements and the other inclusion and exclusion criteria from the parent questionnaires before or after the measurements. The TD children were recruited from schools at Espoo and Kirkkonummi either by inviting the children to participate after a lecture where they had been informed about the study or by sending a recruitment letters to the parents via schools’ electronic message board. Also for the TD group, the eligibility was initially probed on the first phone call or email with the parent, and later controlled from the parent questionnaires, where the parents were asked to list any diagnoses of their child. The study was reviewed and approved by the Ethics Committee of the Helsinki University Hospital. All participants gave their informed consent according to the Declaration of Helsinki. All participants were compensated with two movie tickets.

To gather information on any possible major concurrent comorbid psychiatric or neuropsychiatric conditions, the children in the ADHD group and their caretakers were interviewed with suitable modules (A, C, D, E, F, G, H, I, J, K, N, O, P, Q, R, U, W, and X) from the Finnish version of the diagnostic instrument MINI-KID Interview for Children and Adolescents 7.0 (Sheehan et al., 1998). In this interview, all but two children in the ADHD group met the ADHD diagnostic criteria. Furthermore, three children met the diagnostic criteria for conduct disorder (F91.1), four children for oppositional defiant disorder (F91.3), one child for obsessive-compulsive disorder (F42.8), one child for provisional tic disorder (F95.0), and one child for Tourette’s disorder (F95.2) in the MINI-KID interview. These children were nevertheless included in the study, since the exclusion and inclusion criteria had been met in a recent comprehensive medical examination by experienced child psychiatrists/neurologists.

For their ADHD symptoms, 28 ADHD participants had a methylphenidate prescription, one had a lisdexamfetamine prescription, one had an atomoxetine prescription, and eight were unmedicated. The medication was not taken on the measurement days (24-hour washout period). In addition, six ADHD participants had other ongoing medication (two risperidone prescriptions for behavioral problems, one cetirizine prescription for allergy, two montelukast prescriptions for asthma, one salbutamol prescription for asthma, one melatonin prescription for sleeping problems).

### EPELI Task

EPELI (link to a video) was designed with equal contribution by ML, JS, and ES based on similar previous studies in other patient groups (e.g., Rand et al., 2009; Rendell & Craik, 2000; Shallice & Burgess, 1991). Implementation of the game was conducted by the Peili Vision Company (http://www.peilivision.fi/). An Oculus Go HMD (2560 × 1440 resolution, 60/72 Hz refresh rate, and 101-degree field of view) and its hand controller were used for playing the game, while the experimenter monitored task performance using a Samsung Galaxy Tab S3 tablet. Navigating in the environment was conducted by pointing at a waypoint circle on the floor with a hand controller and simultaneously pressing a button, which resulted in teleporting to that waypoint. Participants used the same button for interacting with the objects. During game play, motion tracking sensors in the goggles as well as in the controller captured the participants’ movements.

The VR environment in EPELI is an apartment that has a children’s room, living room, kitchen, open adult bedroom, utility room, and toilet/bathroom (see Supplemental Methods for the floor plan). In the game, children perform 13 short everyday scenarios. Before the actual game begins, there is a practice session where the participants practice navigating in the environment, interacting with the objects, and monitoring time by using a watch that becomes visible when the participant looks down to the controller and turns its face toward him/herself. A cartoon dragon character in the game guides the child through the practice session and returns to give instructions for each task scenario. Before each task scenario, the dragon gives orally a list of subtasks to be conducted (e.g., put your clothes on, eat breakfast, brush your teeth). Presentation of the 13 task scenarios was counterbalanced so that every other participant conducted them in reversed order. Each task scenario includes four to six subtasks (four subtasks in the task scenarios at the beginning and the end) covered by instructions of 30 to 66 words. In total, there are 70 tasks, 52 of which can be completed at any time, 13 to be completed at a certain time (time-based tasks), and 5 after an external cue (a certain sound, such as doorbell or cell phone tone; event-based tasks). The child is instructed to complete the subtasks in the given order, except for the time- and event-based subtasks, but the completion order does not affect the scoring. One task scenario lasts maximum of 90 seconds but ends earlier if all subtasks are correctly performed. Seven (for participants conducting the task scenarios in forward order) or six (for participants conducting the task scenarios in reverse order) task scenarios are embedded with auditory (dog barking, child coughing, traffic noises, music coming from the radio), as well as audiovisual (fly buzzing nearby the character, tap left running, TV program) distractors. In addition, these conditions contained more task-irrelevant objects. The distracted conditions were counterbalanced across the participants at the same time when the order of the task sets was changed. Distractors were on during the whole task set in the distracted conditions (except the running tap, TV, and music that the participant could switch off). Total duration of EPELI is approximately a maximum of 35 minutes. After the EPELI session, the participants performed Repetition task where they verbally repeated instructions similar to those that the dragon gave in EPELI (eight sentences with a length of 18–54 words). This task assessed the role of the memory component in EPELI performance, as the prospective memory paradigm called for keeping the instructions in mind.

### Parent and Self-Ratings

Parents evaluated their child’s ADHD symptoms, possible executive functions deficits, and possible psychiatric symptoms using the ADHD Rating Scale-IV (ADHD-RS; DuPaul, 1998), the Behavior Rating Inventory for Executive Functions (BRIEF; Gioia et al., 2000), and the Child Behavior Checklist (CBCL; Achenbach, 1991). For description for selecting the dependent variables see Supplemental Methods. To query problems in the specific scenarios presented in EPELI, we designed a new parent questionnaire, the Executive Questionnaire of Everyday LIfe (EQELI; see Supplemental Table 4). To review the experiences of the participants and to acquire information about potential confounds, participants answered to a shortened version of the Presence Questionnaire 3.0 (Witmer et al., 2005), the Simulator Sickness Questionnaire (Kennedy et al., 1993), a gaming experience questionnaire, and an object familiarity questionnaire after playing EPELI (see Supplemental Tables 5 and 6). The child’s familiarity with the tasks was assessed by asking the question “From a scale of 1 to 7, how much have you performed similar tasks in real life?”

#### Conventional neuropsychological tasks

The conventional neuropsychological tasks included in the study included the Similarities and Matrix reasoning subtests of the Finnish version of the Wechsler Intelligence Scale for Children (WISC-IV; Wechsler, 2003), Continuous Performance Task (CPT; Rosvold et al., 1956), Simple Reaction Task (SRT; see Psycho-Motor Vigilance Task in Wilson et al., 2010), Cruiser Task (Kliegel et al., 2013; see also CyberCruiser in Kerns & Price, 2001), Frogs and Cherries Task (F&C; see Dots & Triangles in Zuber et al., 2019), and Heidelberger Exekutivfunktionsdiagnostikum Task (HEXE; Kliegel et al., 2006). Furthermore, for this study we developed Clock Task similar to the finger-snapping task in Kerns and Price (2001). For description of these tasks and the dependent variables, see Supplemental Methods.

### Procedure

Measurements of the TD participants were conducted either in dedicated rooms in schools or at the university facilities (Aalto Behavioral Laboratory, ABL). ADHD participants were measured at university (ABL or the Åbo Akademi University), apart from one participant who was measured at school. Each participant underwent two measurement sessions lasting about 60 minutes each. The first session comprised WISC-IV Matrix Reasoning, EPELI and its related questionnaires (simulator sickness, gaming background, familiarity of the tasks, presence, and object naming questionnaires), and the Repetition task, always in the same order. EPELI was played while in a chair that rotated 360° to help the participants in turning in the game easily and safely. Before starting the game, head set position and sound loudness level were adjusted if needed. The second session comprised WISC-IV Similarities, WISC-IV Digit Span, the clock task, and computerized tasks (CPT, SRT, F&C, HEXE, Cruiser). The order of CPT, SRT, F&C, and the Cruiser tasks was counterbalanced using a Latin square design to control for possible fatigue, while the other tasks were performed at fixed positions in the task battery. The WISC-IV Digit Span was always performed between Cruiser’s practice and experimental phases. Thus, one possible task order was WISC-IV Similarities, SRT, the second trial of the Clock Task, CPT, the third trial of the Clock Task, HEXE practice phase, F&C, HEXE repetition of the plan, the fourth trial of the Clock Task, Cruiser practice phase, WISC-IV Digit Span, Cruiser experimental phase, and HEXE execution phase. The two sessions were conducted either on the same day (separated by a break of at least 15 minutes) or on separate days. The MINI-KID interview was conducted after the second session, preceded by a break of at least 15 minutes.

### Statistical Analyses

All analyses and data visualizations were conducted in *R* version 4.0.3 (R Core Team, 2020) using packages BayesFactor (Morey & Rouder, 2015), pROC (Robin et al., 2011), MatchIt (Ho et al., 2011), psych (Revelle, 2020), rstatix (Kassambara, 2020), lmerTest (Kuznetsova et al., 2017), tidyverse (Wickham et al., 2019), and ggplot2 (Wickham, 2016).

A small part (2.4%) of the task performance data was lost due to technical failures and because of one participant with ADHD refusing to perform the three remaining tasks in the second measurement session. In the following analyses, first, two participants (one from the control group and one from the ADHD group) were removed from the Cruiser task due to purposefully crashing into other cars instead of avoiding them because they felt that this was more fun than the task they had been given. Second, any participants who were not able to repeat the prospective memory task instructions after Cruiser, HEXE or the Clock task were excluded from the corresponding analysis. Third, any possible participants performing near chance level (60% or less on total correct answers) in CPT, F&C, or HEXE were removed from analyses of that task. Fourth, all univariate outliers (±3 SD’s from the group mean) in the dependent variables of the main analysis were excluded. Fifth, the data was checked for possible multivariate outliers (Mahalanobis distance χ^2^ using alpha level *p* < .001) in the dependent variables but none were found. In total, the amount of data removed was 9.0%.

For EPELI, we operationalized several indices and scores that reflect task performance and task-related behavior (see Supplemental Table 1). For the event-based subtasks, only those completed within 10 seconds after the target event were treated as correct. For the time-based subtasks, only those which were completed within 10 seconds before or after the target time were taken as correct. The final number of variables was reduced by examining their pairwise correlations in the control group and removing one variable from each pair when the correlation was .85 or more. The variables remaining after this procedure included Total score (correctly performed subtasks), Task efficacy (percentage of relevant actions, that is, actions that were necessary to perform any successfully completed subtask, out of all actions excluding clicks on the waypoints that enable moving around in the environment), Navigation efficacy (Total score divided by distance covered, which includes distance walked and the distance to each manipulated object at the time they were clicked), Controller motion (controller angular movement during task performance), and Total actions (number of clicks plus number of times hitting the drums in the children’s room by swinging the controller, also including the clicks during the instruction phase of each task scenario), time-based subtask score, number of clock checks, and event-based subtask score. Of these variables, the first five were regarded as the main variables (see Table 1) and the last three represented a secondary set related to specific aspects of prospective memory. Since we did not make any separate pre-registered hypotheses concerning time- or event-based tasks in EPELI, only the first five main EPELI measures were analyzed here.

Group differences on the background variables were tested with *t*-tests and Fisher’s exact test. To examine the *predictive validity* of the EPELI measures, the effects of group (ADHD/control) and distractors (on/off) on the EPELI variables were tested using two-way analysis of variance. Furthermore, three-way analysis of variance with playing order as the third independent variable was performed, but as these analyses yielded very similar results, those results are not shown. The effects of scenario-to-scenario task progression on EPELI variables were examined with linear mixed models. Based on Bayesian Information Criterion, random intercept model was the best fitting error covariance structure for all dependent variables. The scenario-to-scenario variabilities of the EPELI variables were tested using *t*-tests for group effects. The group differences on the conventional neuropsychological tasks were examined using *t*-tests. Furthermore, Bayes factors were calculated for all the effects mentioned above. Based on visual inspection, all the assumptions of analysis of variance were met.

*Discriminant validity* of each EPELI variable was assessed by calculating the area under curve (AUC) from the receiver operating characteristic (ROC) curve. A cutoff point with the highest percentage of correctly classified cases was determined by Youden’s index and the sensitivity and specificity of the variable at this cutoff was reported. Similar analyses were performed for the conventional neuropsychological tests. To evaluate the multivariate classification capacity of EPELI and CPT, logistic regression was applied separately to the main dependent variables of each test, and the classification value of the resulting variables were examined the same way.

*Concurrent validity* was assessed by calculating Pearson’s correlation coefficients over all participants between the EPELI variables and questionnaires. Similar correlation analyses were performed between the EPELI measures and conventional neuropsychological tests, as well as between the latter measures and the questionnaires, but these were considered as secondary analyses.

## Results

### Behavioral Characteristics

The background characteristics of the participants are presented in Table 2. The ADHD and TD groups did not differ in terms of age, handedness, gender, parental income, parental education, verbal reasoning abilities, or perceptual reasoning abilities. Parents rated more inattention and hyperactivity-impulsivity symptoms (ADHD-RS) and everyday attention and executive function problems (BRIEF) for the ADHD children than for the TD children. The ADHD children also had a higher number of internalizing and externalizing symptoms, as indicated by CBCL. Parents of the ADHD children also reported more difficulties than parents of the TD children in real-life situations that we simulated in EPELI, as indicated by our EQELI questionnaire (see Supplemental Table 4). There were no group differences in the gaming experience, perceived familiarity of the tasks, or overall presence experiences. Both groups were able to reliably name the objects that were included in EPELI (object naming task), which was taken to reflect that they were familiar with the vocabulary used in the game. The participants reported very few negative experiences in the simulator sickness questionnaire.

### Predictive and Discriminant Validity of the EPELI Measures

#### Predictive validity analyses.^1^

**Figure 1.**
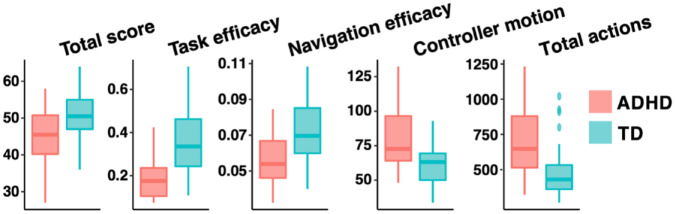
Boxplots showing the minimum, maximum, sample median, and first and third quartiles, as well as the potential outliers (±1.5 × interquartile range) for main EPELI measures in the ADHD and TD group.

The box plots of EPELI measures per group are presented in Figure 1 and the results of the analysis of variance are presented in Table 3. For the Total score, there were main effects of group and distractions, with the TD group having higher scores than the ADHD group and the non-distracted task scenarios yielding higher scores than the distracted ones. Task efficacy and Navigation efficacy revealed main effects of group and distractions, with the TD group being more efficient than the ADHD group and efficacy being higher in the non-distracted task scenarios than in the distracted ones. For Controller motion, there were again main effects of group and distractions: the ADHD group moved more than the TD group, and there was more motion in the distracted than in the non-distracted task scenarios. For Total actions, there was a main effect of group, with the ADHD group having higher rates of actions than the TD group.

**Table 3.** Analysis of Variance for the Effects of Group, Distractions, and Group × Distraction Interaction for the Main EPELI Measures.

Dependent variable	Effect	*F*(1, 74)	*p*	η^2^_G_	1/BF
Total score	Group	12.31	<.001	.12	39.03
	Distractions	26.97	<.001	.07	8,550.36
	Group × distractions	2.50	.118	.01	0.60
Task efficacy	Group	35.64	<.001	.28	145,317.90
	Distractions	16.31	<.001	.04	167.64
	Group × distractions	0.113	.738	<.01	0.25
Navigation efficacy	Group	16.67	<.001	.16	205.41
	Distractions	31.13	<.001	.07	34,542.69
	Group × distractions	1.51	.223	<.01	0.45
Controller motion	Group	16.79	<.001	.16	219.42
	Distractions	6.00	.017	.01	2.58
	Group × distractions	1.76	.188	<.01	0.51
Total actions	Group	19.75	<.001	.19	579.12
	Distractions	0.30	.585	<.01	0.21
	Group × distractions	0.04	.840	<.01	0.24

*Note*. BF = Bayes Factor.

Regarding scenario-to-scenario progression (see Supplemental Figure 1), there was a main effect of time on Task efficacy (*t*[910] = −5.61, *p* < .001), Navigation efficacy (*t*[910] = −4.19, *p* < .001), Controller motion (*t*[910] = 3.92, *p* < .001), and Total actions (*t*[910] = 7.27, *p* < .001). In line with analysis of variance (Table 3), linear mixed models showed a main effect of group in all dependent variables. For three variables, there was also an time × group interaction: on Task efficacy (*t*[910] = 2.21, *p* = .027) and Navigation efficacy (*t*[910] = 2.43, *p* = .015) the ADHD group showed stronger decline, and on Total actions (*t*[910] = −3.05, *p* < .002) the ADHD group exhibited stronger increase over time. There were group differences also in the scenario-to-scenario variability (SD) of Task efficacy (*t*[74] = −3.67, *p* < .001), with the TD group demonstrating more variability. Since Task efficacy is the percentage of relevant actions out of total actions excluding moving actions, a separate analysis for the variabilities of its constituent measures was conducted to be able to interpret the variability in Task efficacy. There was no group difference in the number of relevant actions, but total actions excluding moving actions yielded a group difference with the ADHD group demonstrating more variability (*t*[74] = 3.53, *p* < .001). Thus, the group difference in Task efficacy is caused by more variability in total actions in the ADHD group. Furthermore, the ADHD group demonstrated more variability in Controller motion (*t*[74] = 4.10, *p* < .001) and Total actions (*t*[74] = 3.53, *p* < .001). There were no group differences in Total score or Navigation efficacy variability.

#### Discriminant validity analyses

**Figure 2.**
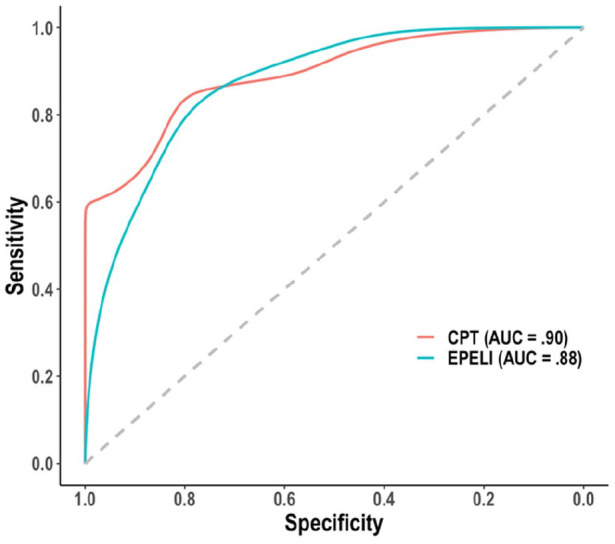
ROC curves for the logistic regression analyses with five EPELI and CPT measures.

The AUCs and cutoff values based on Youden’s index for the main EPELI measures, as well as the logistic regression utilizing all five variables at the same time, are presented in Table 4. Of the single EPELI variables, Task efficacy has the highest AUC point estimate (.83). The multi-measure logistic regression analysis (see Figure 2 for the ROCs) yielded slightly higher AUC point estimate (.88), but the difference from the AUC of Task efficacy was not significant.

**Table 4.** Areas Under the Curve (AUCs) From Receiver Operating Characteristic Curve Analyses, 95% Confidence Intervals and Optimal Cutoffs for Each EPELI Measure Analyzed Separately and for Logistic Regression Analysis Utilizing all the Measures at the Same Time.

Variable	AUC	95% CI	Optimal cutoff^a^		Specificity (%)
			Threshold	Sensitivity (%)	
Total score	0.70	0.59–0.82	46.5	76	55
Task efficacy	0.83	0.74–0.92	0.29	66	89
Navigation efficacy	0.75	0.64–0.86	0.06	76	66
Controller motion	0.73	0.62–0.85	68,588.85	71	66
Actions	0.78	0.68–0.89	463	61	89
Logistic regression analysis	0.88	0.80–0.94	0.431	79	87

a Based on Youden’s index.

### Group Differences and Discriminative Ability of the Conventional Neuropsychological Tasks

**Figure 3.**
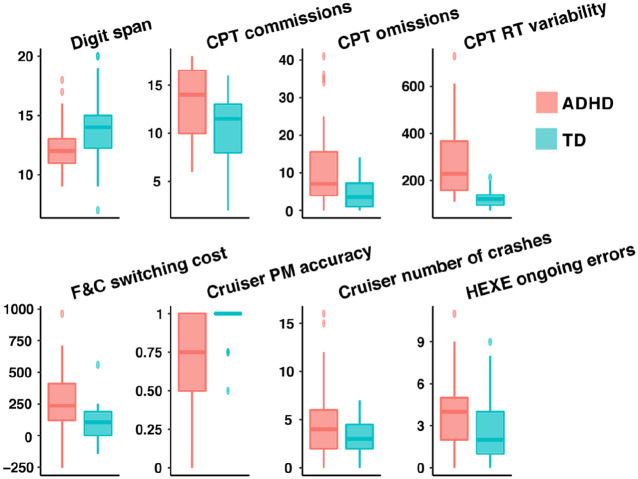
Boxplots for the conventional neuropsychological measures showing group differences.

Table 5 shows the group means, standard deviations and test statistics of the conventional neuropsychological tasks, and the distributions of task variables with significant group differences are depicted in Figure 3. The TD group performed better than the ADHD group in the Digit span task but not in the Repetition task, where the material was akin to the instructions heard during EPELI. As regards CPT, the ADHD group made more omission and commission errors and had higher variability in reaction time than the TD group. The ADHD group also showed longer mean reaction times in SRT. Moreover, the ADHD group demonstrated a higher switching cost in the F&C task. With regard to prospective memory tasks, the ADHD group performed worse than the TD group in the Cruiser, which tapped on time-based prospective memory, but there was no group difference in the Clock task or on the HEXE task prospective memory measures (self-initiation and switching). Regarding ongoing task performance, the ADHD group made more mistakes than the TD group both in the Cruiser (number of crashes) and HEXE (ongoing errors) tasks, even though there was no difference in the number of correct ongoing task responses in HEXE. Regarding time monitoring in the Cruiser task, the TD group checked the time more often than the ADHD group.

**Table 5.** Test Statistics for the Conventional Neuropsychological Tasks.

Variable	Test statistic	*p*	1/BF	Effect size^a^
Digit span	*t*(74) = −2.78	.007	6.14	0.638
Repetition task	*t*(74) = 1.04	.301	0.38	0.239
CPT omissions	*t*(65) = −3.16	.002	14.80	−0.786
CPT commissions	*t*(65) = −2.91	.005	8.08	−0.711
CPT RT variability	*t*(65) = −5.54	.000	21755.35	−1.38
SRT mean RT	*t*(73) = −2.98	.004	9.62	−0.684
F&C switching cost	*t*(66) = −3.21	.002	16.64	−0.765
Cruiser PM accuracy	*t*(70) = 3.83	<.001	91.93	0.913
Cruiser monitoring	*t*(70) = 2.23	.029	1.98	0.527
Cruiser number of crashes	*t*(70) = −2.48	.016	3.20	−0.589
Clock task PM accuracy	*t*(72) = 0.47	.637	0.25	0.11
HEXE correct task responses	*t*(57) = −1.31	.195	0.54	−0.335
HEXE ongoing errors	*t*(57) = −2.08	.028	1.57	−0.544
HEXE self-initiated PM task	Fisher’s exact test	.332	0.64	0.094
HEXE switching PM task	Fisher’s exact test	.691	0.51	<0.001

*Note.* PM = prospective memory; RT = reaction time; BF = Bayes factor.

a Cohen’s *d* for continuous variables, Cramér’s *V* for categorical variables.

The AUCs and cutoff values based on Youden’s index for the conventional neuropsychological tasks and for the logistic regression analysis utilizing all five CPT variables at the same time are presented in Table 6. The highest AUC point estimate (.90) is yielded by the logistic regression analysis, but this is this not significantly higher (*p* > .05) than the AUC point estimate for CPT RT variability (.85). Considering the AUC estimates of EPELI and the conventional neuropsychological tasks together, the highest estimates are those of EPELI logistic regression analysis, EPELI Task efficacy, CPT logistic regression analysis, and CPT RT variability, which did not differ from each other (*p* > .05). AUC obtained from EPELI Task efficacy was significantly higher than in most of the conventional neuropsychological tasks, except Digit span, CPT RT variability, F&C switching cost, and HEXE ongoing errors.

**Table 6.** Areas Under the Curve (AUCs) From Receiver Operating Characteristic Curve Analyses, 95% Confidence Intervals and Optimal Cutoffs for Traditional Neuropsychological Tasks.

Variable	AUC	95% CI	Optimal cutoff^a^		
			Threshold	Sensitivity (%)	Specificity (%)
Digit span	0.70	0.58–0.82	12.5	74	66
Repetition task	0.47	0.34–0.60	25.5	39	66
CPT omission errors	0.70	0.57–0.82	3.5	50	80
CPT commission errors	0.70	0.58–0.82	13.5	78	51
CPT RT variability	0.85	0.76–0.94	150.32	88	77
SRT mean RT	0.67	0.54–0.79	508.12	58	73
F&C switching cost	0.71	0.58–0.84	274.01	97	47
Cruiser PM accuracy	0.70	0.60–0.80	0.88	86	51
Cruiser number of clock checks	0.66	.53–.78	18.5	77	54
Cruiser number of crashes	0.63	0.50–0.76	7.5	100	22
Clock task PM accuracy	0.48	0.36–0.60	0.0	0	100
HEXE correct task responses	0.55	0.38–0.72	41	82	44
HEXE ongoing errors	0.67	0.53–0.81	2.5	62	72
CPT logistic regression analysis	0.90	0.82–0.96	0.43	81	86

*Note.* RT = reaction time.

a Based on Youden’s index.

### Concurrent Validity of the EPELI Measures

The correlations of EPELI with the BRIEF and the ADHD-RS questionnaires across all participants that were used to examine concurrent validity are presented in Table 7. All EPELI measures correlated with both BRIEF and ADHD-RS (range *r* absolute value = .312–.574). For EPELI’s Total score, Task efficacy, and Navigation efficacy, this correlation is negative, so that higher performance in these measures is associated with fewer problems with executive function and lower ADHD symptom scores. For Controller motion and Total actions, the direction is the opposite.

**Table 7.** Correlations between the main EPELI measures and conventional neuropsychological task measures and BRIEF and ADHD-RS scales.

Variable	BRIEF		ADHD-RS	
	*r*	95% CI	*r*	95% CI
EPELI total score	−.356**	[−.539, −.142]	−.312*	[−.502, –.093]
EPELI task efficacy	−.574***	[−.708, −.400]	−.553***	[−.692, –.375]
EPELI navigation efficacy	−.466***	[−.626, −.269]	−.453***	[−.615, −.253]
EPELI controller motion	.414***	[.208,.585]	.43***	[.227,.598]
EPELI total actions	.457***	[.258,.619]	.477***	[.282,.634]
Digit span	−.304*	[−.496, −.084]	−.249	[−.449, −.024]
Repetition task	−.144	[−.358,.084]	−.057	[−.279,.171]
CPT omissions	.368**	[.141,.559]	.38**	[.153,.568]
CPT commissions	.329*	[.096,.527]	.353**	[.123,.546]
CPT variability	.476***	[.266,.643]	.469***	[.258,.638]
SRT mean RT	.372**	[.159,.553]	.335**	[.116,.522]
F&C switching cost	.219	[−.020,.435]	.175	[−.066,.397]
Cruiser PM accuracy	−.329*	[−.521, −.106]	−.288*	[−.487, −.061]
Cruiser monitoring	−.213	[−.424,.019]	−.18	[−.395,.054]
Cruiser number of crashes	.232	[.000,.440]	.146	[−.089,.365]
Clock task PM accuracy	−.042	[−.268,.189]	−.033	[−.260,.197]
HEXE correct task responses	.225	[−.033,.455]	.227	[−.031,.456]
HEXE ongoing errors	.257	[.001,.481]	.267	[.012,.490]
HEXE self-initiated PM task	.017	[−.238,.270]	.073	[−.184,.321]
HEXE switching PM task	−.064	[−.195,.315]	−.027	[−.281,.231]

*Note.* FDR adjusted point estimates with unadjusted 95% confidence intervals.

* *p* ≤ .05. **p ≤ .01. ***p ≤ .001.

### Associations Between Conventional Neuropsychological Tasks and Parent-rated Executive Deficits and ADHD Symptoms

Table 7 also includes the correlations of conventional neuropsychological tasks with the BRIEF and the ADHD-RS questionnaires across all participants. Regarding conventional neuropsychological tasks, the range of the absolute values of the correlations to BRIEF and ADHD-RS was .017 to .476. CPT measures and SRT reaction time yielded positive correlations for both questionnaires, whereas Digit Span correlated negatively with BRIEF and prospective memory accuracy in Cruiser with both BRIEF and ADHD-RS. The correlation between EPELI Task efficacy and BRIEF was stronger than any of the correlations between conventional neuropsychological tests and BRIEF, except CPT RT variability (uncorrected *p* < .05). In the FDR corrected statistics, there were also no differences between the correlations of EPELI Task efficacy and BRIEF correlation versus the correlations between of BRIEF and CPT omission errors, CPT commission errors, and SRT mean RT.

### Associations Between EPELI Measures and Conventional Neuropsychological Tasks

Table 8 shows the correlations between the main EPELI measures and those conventional neuropsychological measures that yielded group differences. CPT commission errors were positively correlated with Controller motion and Total actions in EPELI. CPT omissions exhibited a negative correlation with EPELI Task and Navigation efficacy, and a positive correlation with Total actions in EPELI. CPT RT variability was negatively correlated with EPELI efficacy measures and positively correlated with Controller motion and Total actions. Regarding SRT mean RT, a negative correlation to EPELI Total score and EPELI Task and Navigation efficacies was found. Also, the switching cost in F&C was negatively correlated with Total score in EPELI. The prospective memory performance in the Cruiser task showed positive correlation with EPELI Total score, Task efficacy, and Navigation efficacy. Furthermore, the HEXE ongoing task performance (ongoing errors) was correlated with all EPELI measures except the Total score.

**Table 8.** The Correlations Between the Main Epeli Measures and Those Conventional Neuropsychological Measures That Yielded Significant Group Differences.

	Digit span	CPT commissions	CPT omissions	CPT RT variability	SRT mean RT	F&C switching cost	Cruiser PM accuracy	Cruiser number of crashes	HEXE ongoing errors
Total score	.244	−.151	−.181	−.252	−.383**	−.315*	.452***	−.110	−.354*
Task efficacy	.167	−.260	−.346*	−.407**	−.278*	−.218	.338*	−.135	−.515***
Navigation efficacy	.211	−.254	−.290*	−.351*	−.331*	−.220	.358**	−.061	−.425**
Controller motion	−.134	.295*	.193	.294*	.072	.115	−.192	−.001	.566***
Total actions	−.171	.312*	.375**	.413**	.220	.093	−.197	.071	.581***

*Note.* FDR Correction.

* *p* ≤ .05. **p ≤ .01. ***p ≤ .001.

The correlations between the main EPELI measures and conventional neuropsychological measures not yielding group differences are presented in Supplemental Table 3. The Repetition task was associated with all EPELI measures correlating positively with Total score, Task efficacy and Navigation efficacy in EPELI. Both reasoning subtests (Similarities and Matrix reasoning) from WISC-IV correlated positively with EPELI Total score, but Similarities correlated also with both EPELI efficacy measures. Cruiser monitoring was positively associated with EPELI Total score, while the total number of correct task responses in HEXE shared a positive correlation with Controller motion and Total actions in EPELI.

## Discussion

Neurodevelopmental disorders such as ADHD do not fall into categorical cognitive domains (Willcutt et al., 2005), but rather manifest themselves as heterogeneous phenotypes with idiosyncratic behavioral characteristics (Luo et al., 2019). We developed a novel naturalistic paradigm named as EPELI that aimed to objectively characterize attentional-executive dysfunction in a complex open-ended condition, and tested it in a pre-registered hypothesis-driven study with a group of ADHD children and TD controls in VR using an HMD. Our main aim was to test the predictive, discriminant and concurrent validity of the EPELI task by examining the group differences and ROC characteristics of its main measures and their associations with parent-rated ADHD symptoms and executive function deficits. Supporting the predictive validity of EPELI and our hypothesis that ADHD children perform worse in EPELI than TD controls, all five main EPELI measures, operationalized to reflect attentional-executive deficits and hyperactivity-impulsivity, showed the expected group differences (Table 3, Figure 1). Furthermore, EPELI showed discriminant validity as the multiple logistic regression analysis with the five EPELI measures had an excellent AUC of 88% (Table 4, Figure 2). EPELI’s concurrent validity was also confirmed, as the EPELI measures were correlated with parent-evaluated everyday executive functioning and ADHD symptoms (Table 7). Out of the five main EPELI measures, Task efficacy showed the most clear-cut of group difference, the highest classification accuracy, and the highest correlations with parent-evaluated everyday attention deficits and symptoms. On the other hand, we did not find support for our hypothesis that the distractor effect would be larger for the ADHD children than for the typically developing children. Also, the scenario-to-scenario changes were different than expected.

### EPELI Measures Reflecting the ADHD Core Symptomatology

The five EPELI measures selected for the final analysis were assumed to reflect ADHD-related symptoms and key aspects in executive functions required in the task. As regards to predictive validity, the expected group differences were present in all five measures (Table 3, Figure 1). According to a multiple regression analysis using all these five measures, the discriminant validity of EPELI was excellent and comparable to that of CPT, the current gold standard in ADHD assessment (see Albrecht et al., 2015; Ogundele et al., 2011). This is certainly a promising result, given the long-standing problems in finding ADHD test measures with a high discriminative power. This result is not due to a lower performance of our CPT version either: the present AUC of 90% for CPT is on the higher side when compared to other studies, suggesting that our CPT version was functioning well (for a review, see Huang-Pollock et al., 2012).

Out of the five selected EPELI measures, Task efficacy was a particularly important variable in the present analyses. Representing the relative percentage of relevant actions out of all actions, it is related to selective attention, which is typically defined as focusing on a target object while not reacting to irrelevant ones. However, while traditional attention measures often address a specific attentional component in a simplified context, this EPELI measure covers various aspects of the participant’s interactions with the environment (listening to the instructions and keeping those in mind during the task, planning how to perform the list of tasks, executing the tasks, monitoring own performance), coming closer to the diagnostic definitions of inattention as it manifests itself in everyday life. The closer match to the diagnostic definitions was expected to boost the predictive validity of EPELI Task efficacy and result in stronger correlations with the subjective questionnaires than what is seen with conventional neuropsychological tasks, and such findings were indeed observed. Our global inattention measure showed robust group differences, was informative in predicting the group status of individual participants and was strongly associated with ADHD symptoms and everyday EF dysfunction.

In the everyday life situations that EPELI attempts to simulate, hyperactivity and impulsivity may be present in the same situations as inattention but have different behavioral manifestations. Hyperactivity is a relatively straightforward symptom to measure, as it is largely related to the physical movement of the individual. In previous studies, activity levels of ADHD participants have been quantified using various sensor technologies (see De Crescenzo et al., 2016 for a meta-analysis). Naturalistic motion tracking studies face the challenge of controlling for contextual effects and distinguishing abnormal or non-adaptive motion patterns from typical overall activity levels. There are studies that also register participant motion during cognitive tasks (e.g., Teicher et al., 1996), but they usually include tasks where constant inhibition of movement is desired, even though movement is an integral part of everyday life. In contrast, EPELI hyperactivity measures index typical spontaneous behavior in naturalistic situations. Our results demonstrate that ADHD children clearly display excessive overall controller motion and controller motion variability compared to TD peers (Figure 1, Supplemental Table 2). Previously, it has been suggested that hyperactivity would be most clearly observed in cognitive tasks where the level of stimulation is low (Kofler et al., 2016). While this may well be the case, we provide new evidence that hyperactivity in ADHD participants can also be objectively measured in lifelike situations where the participants are moving freely. Thus far, the focus has mostly been on head movements (see, e.g., Mangalmurti et al., 2020; Parsons et al., 2019). We selected controller motion as the hyperactivity measure, since it is more closely related to performing actions in the game, whereas head movements can also reflect visual search.

Regarding impulsivity, defined based on Total actions, our hypothesis was that ADHD participants would perform a higher number of actions, trying to impulsively interact with various functional objects in the game (e.g., toys, drums, and TV). The results provided clear support for the hypothesis, further showing that the number of these impulsive actions fluctuated more over time in the ADHD participants more than in TD participants. One can question whether these kinds of impulsive actions triggered by a potential immediate reward would be more representative of the daily problems that ADHD children face than, for instance, the ability to inhibit their response to a non-target letter in a continuous sequence of stimuli (i.e., CPT). Our impulsivity measure bears a greater resemblance to delayed reward tasks where the target that triggers impulsive behavior is motivating (e.g., Dalley & Robbins, 2017). A key aspect to consider here is that in EPELI, impulsive actions carried no penalty, and the measure is therefore assumed to reflect typical spontaneous behavior in an environment where the participants perform volitional actions.

Besides the three measures operationalized based on the ADHD symptoms, two other general attentional-executive EPELI measures were also included. Total score was the number of correctly performed subtasks and Navigation efficacy was another efficacy measure for which the Total score was divided by the distance covered. As the overall task relies on prospective memory, it was expected that Total score would correlate with our control measure of memory (the Repetition task) where the participants simply repeated task lists similar to those that the dragon provided in the game, which indeed was the case (see Supplemental Table 3). Total score resembles the performance measures previously used in naturalistic prospective memory tasks such as the Virtual Multiple Errands Test (Rand et al., 2009). To the best of our knowledge, multitasking measures of this type have not been previously used to assess executive functions in children with ADHD, but there is evidence that such measures can detect executive dysfunction in various other clinical conditions (e.g., Cipresso et al., 2014; Rand et al., 2009). Interestingly, there were no differences between ADHD children and TD controls in how well they recalled the instructions in the Repetition task, suggesting that the lower Total score in ADHD children was more closely related to task execution than to remembering what to do. Further research to develop more ecologically valid measures of time-based prospective memory is certainly needed, as there is evidence that this domain is clearly an important factor in daily life (Haas et al., 2020) and compromised in ADHD (Talbot et al., 2018).

Against our hypothesis, there was practically no improvement in EPELI performance from one task scenario to another in either the ADHD or the TD group as measured by Total score (see Supplemental Figure 1). One explanation for this could be that EPELI tasks are highly familiar and may not prompt within-task strategy development in the same way as novel tasks do (see Gathercole et al., 2019). Interestingly, both Task efficacy and Navigation efficacy evidenced decline during the gameplay, with the ADHD group declining more than the TD group. At the same time, an increase in Controller motion and All actions across the scenarios was observed. This may indicate an increase in hyperactivity-impulsivity symptoms, possibly explained by a decrease in top-down control (e.g., Mangalmurti et al., 2020). As hypothesized, the ADHD group displayed more variability in the Controller motion and All actions measures during the gameplay. In Task efficacy, there was a group effect caused by more variability of total actions in ADHD group. The distractions and extraneous objects resulted in lower Total scores, lower efficacies, and higher Controller motion for both groups, but the hypothesized disproportionate distractor effect in the ADHD group was not observed. One possible explanation for the lack of this interaction effect is that even the non-distracted task scenarios included all kinds of task-irrelevant but tempting objects that may have distracted the ADHD children more than the TD children. This interpretation is supported by the findings that the ADHD children displayed less efficient performance throughout EPELI. It should also be noted that in many of the previous studies distractors have been instantaneous (see Parsons et al., 2019), while in the present study the audiovisual fly distractor as well as the extraneous objects were present during the whole scenario. Distractor effects to constant irrelevant stimuli may be different than for sudden changes in the environment, and it is possible that our distractors were not ideally suited for quantifying distraction in ADHD children.

### Links Between the EPELI Measures, Questionnaires of Everyday Problems, and Conventional Neuropsychological Tasks

Current key challenges in the use of experimental tasks in ADHD diagnostic assessment include the weak correspondence between experimental measures and the symptoms defined in the diagnostic classification system, and the limited predictive power of the experimental measures (Barkley & Murphy, 2010, 2011). Our study provides new behavioral evidence that VR-based simulations of real-life conditions not only distinguish reliably between ADHD participants and TD controls, but also correlate strongly with real-life attentional-executive deficits as measured by questionnaires (see Table 7). Linking experimental measures with the symptoms has also been a major target in previous VR studies of ADHD participants, which in most cases have utilized the virtual classroom setup (see Parsons et al., 2019 for a meta-analysis). We did not perform a direct comparison with these VR-based methods, but EPELI performed well in the comparison with the conventional experimental methods. Specifically, correlation coefficients were clearly higher for EPELI than for conventional experimental tasks and despite the relatively small sample, the differences between the correlations were robust, indicating concurrent validity.

Inclusion of the conventional experimental measures was motivated not only by comparing how well they explain the group status or symptoms in comparison with EPELI, but also by examining to what extent the EPELI measures are linked to these tasks. A few clear associations between the EPELI measures and conventional neuropsychological measures were observed. EPELI efficacy measures were negatively associated with RT variability in CPT. This is interesting because RT variability is one of the rare measures that is not expected to reflect maximal performance, but rather fluctuations of performance over time (e.g., Sonuga-Barke & Castellanos, 2007). Such fluctuations were also present in the EPELI measures, suggesting that naturalistic tasks could be used to study attention dynamics that have also recently been investigated in virtual-classroom studies (e.g., Mangalmurti et al., 2020). Besides attention fluctuations, we found evidence of links between EPELI and conventional measures in the domain of prospective memory. EPELI Total score is essentially a prospective memory measure, and it is thus reasonable that it correlated with another prospective measure stemming from the Cruiser task. Furthermore, the number of errors in the ongoing task of the HEXE prospective memory task was associated with all five EPELI measures. It is possible that indulging in less goal-oriented and more exploratory behavior resulted in lower total score and greater amount of irrelevant action (i.e., lower efficacy) and movement in EPELI, as well as more error-prone performance in HEXE. Overall, further research is needed to clarify the cognitive functions reflected by the EPELI measures. However, such efforts face challenges due to the task impurity issue and discrepancies in the factorial structure of conventional experimental executive function measures (Snyder et al., 2015).

### Limitations of the Present Study

Despite the promising findings, there are several limitations to consider when interpreting our results. As the inter-individual variability in ADHD symptoms is high, a larger sample would be required to attain more robust results that could be more reliably generalized to the general ADHD population. In particular, the results of classification analyses and correlational analyses are influenced by sampling, and these results should be interpreted carefully with the sample size and participant selection criteria in mind. Despite the use of propensity matching and exclusion of several potential confounding factors, there could also be other relevant background factors on which the groups differ. In future studies, more detailed assessment of factors explaining individual variability in EPELI measures should be performed. The sample size also limits the choice of analysis methods. A larger sample would potentially enable one to further separate several important factors, such as the role of specific symptom domains or executive functions. A higher number of participants would also benefit data-driven analyses of VR data (Mangalmurti et al., 2020). Another possible limitation relates to the representativeness of the home environment that was used as the context here. Although home situations play a particularly important role in the diagnostics, it is critical that the symptoms manifest in different contexts. There is evidence that impoverished experimental tasks have limited generalizability to real-life situations, but it is unclear whether simulation of one everyday context predicts behavior in another context. In future studies, other situations and contexts, for instance school day activities, could also be simulated in VR. Moreover, a direct comparison to classroom-based VR-CPT would be useful to further examine the pros and cons of these two approaches. In our study, a conventional version of CPT was used and based on pilot experiments in healthy participants, we decided to shorten this task so that the overall test battery would not be too demanding and lead to attrition problems.

## Conclusions

Our study provides novel behavioral evidence that naturalistic VR is a reliable method to assess and quantify real-life attention and executive function deficits in ADHD. Compared to more classical paradigms, advantages of this approach include opportunities to (a) measure complex behavioral patterns in situations resembling those where the symptoms occur, (b) capture volitional behaviors reflecting typical behavior in open-ended situations that mimic real-life situations more closely, (c) provoke particular symptoms with specific experimental manipulations (e.g., adding attractor stimuli to encourage impulsive actions, placing high attentional demands to capture inattention), and (d) quantifying the natural pace of participant’s motion with sensor technology. Regarding the participants’ experience, using a game-like paradigm with varied tasks and rich stimuli is probably more convenient and less tedious than simplistic tasks with restricted stimuli. Indeed, this assumption is supported by our findings that both ADHD and TD children on average rated playing EPELI as a highly enjoyable experience (see Supplemental Table 6, questions 10 & 11).

The present proof-of-concept study showed that EPELI has predictive validity by differentiating between ADHD children and typically developing controls, discriminant validity by differentiating individual children based on their group status, and concurrent validity by being significantly associated with parent-rated problems in managing situations with high cognitive demands in real life. Besides shedding light on the naturalistic behavior of ADHD children in daily situations, this study opens new avenues for the objective measurement of ADHD symptoms. Taken together, these results suggest that measuring everyday attentional-executive deficits linked to ADHD symptoms is possible with our new EPELI task. We hope that these findings will facilitate the development of naturalistic approaches for the assessment of neurodevelopmental disorders.

## Supplemental Material

sj-pdf-1-jad-10.1177_10870547211044214 – Supplemental material for Quantifying ADHD Symptoms in Open-Ended Everyday Life Contexts With a New Virtual Reality Task Click here for additional data file. Supplemental material, sj-pdf-1-jad-10.1177_10870547211044214 for Quantifying ADHD Symptoms in Open-Ended Everyday Life Contexts With a New Virtual Reality Task by Erik Seesjärvi, Jasmin Puhakka, Eeva T. Aronen, Jari Lipsanen, Minna Mannerkoski, Alexandra Hering, Sascha Zuber, Matthias Kliegel, Matti Laine and Juha Salmi in Journal of Attention Disorders

sj-pdf-2-jad-10.1177_10870547211044214 – Supplemental material for Quantifying ADHD Symptoms in Open-Ended Everyday Life Contexts With a New Virtual Reality Task Click here for additional data file. Supplemental material, sj-pdf-2-jad-10.1177_10870547211044214 for Quantifying ADHD Symptoms in Open-Ended Everyday Life Contexts With a New Virtual Reality Task by Erik Seesjärvi, Jasmin Puhakka, Eeva T. Aronen, Jari Lipsanen, Minna Mannerkoski, Alexandra Hering, Sascha Zuber, Matthias Kliegel, Matti Laine and Juha Salmi in Journal of Attention Disorders

sj-pdf-3-jad-10.1177_10870547211044214 – Supplemental material for Quantifying ADHD Symptoms in Open-Ended Everyday Life Contexts With a New Virtual Reality Task Click here for additional data file. Supplemental material, sj-pdf-3-jad-10.1177_10870547211044214 for Quantifying ADHD Symptoms in Open-Ended Everyday Life Contexts With a New Virtual Reality Task by Erik Seesjärvi, Jasmin Puhakka, Eeva T. Aronen, Jari Lipsanen, Minna Mannerkoski, Alexandra Hering, Sascha Zuber, Matthias Kliegel, Matti Laine and Juha Salmi in Journal of Attention Disorders

sj-pdf-4-jad-10.1177_10870547211044214 – Supplemental material for Quantifying ADHD Symptoms in Open-Ended Everyday Life Contexts With a New Virtual Reality Task Click here for additional data file. Supplemental material, sj-pdf-4-jad-10.1177_10870547211044214 for Quantifying ADHD Symptoms in Open-Ended Everyday Life Contexts With a New Virtual Reality Task by Erik Seesjärvi, Jasmin Puhakka, Eeva T. Aronen, Jari Lipsanen, Minna Mannerkoski, Alexandra Hering, Sascha Zuber, Matthias Kliegel, Matti Laine and Juha Salmi in Journal of Attention Disorders

sj-pdf-5-jad-10.1177_10870547211044214 – Supplemental material for Quantifying ADHD Symptoms in Open-Ended Everyday Life Contexts With a New Virtual Reality Task Click here for additional data file. Supplemental material, sj-pdf-5-jad-10.1177_10870547211044214 for Quantifying ADHD Symptoms in Open-Ended Everyday Life Contexts With a New Virtual Reality Task by Erik Seesjärvi, Jasmin Puhakka, Eeva T. Aronen, Jari Lipsanen, Minna Mannerkoski, Alexandra Hering, Sascha Zuber, Matthias Kliegel, Matti Laine and Juha Salmi in Journal of Attention Disorders

sj-pdf-6-jad-10.1177_10870547211044214 – Supplemental material for Quantifying ADHD Symptoms in Open-Ended Everyday Life Contexts With a New Virtual Reality Task Click here for additional data file. Supplemental material, sj-pdf-6-jad-10.1177_10870547211044214 for Quantifying ADHD Symptoms in Open-Ended Everyday Life Contexts With a New Virtual Reality Task by Erik Seesjärvi, Jasmin Puhakka, Eeva T. Aronen, Jari Lipsanen, Minna Mannerkoski, Alexandra Hering, Sascha Zuber, Matthias Kliegel, Matti Laine and Juha Salmi in Journal of Attention Disorders

sj-pdf-7-jad-10.1177_10870547211044214 – Supplemental material for Quantifying ADHD Symptoms in Open-Ended Everyday Life Contexts With a New Virtual Reality Task Click here for additional data file. Supplemental material, sj-pdf-7-jad-10.1177_10870547211044214 for Quantifying ADHD Symptoms in Open-Ended Everyday Life Contexts With a New Virtual Reality Task by Erik Seesjärvi, Jasmin Puhakka, Eeva T. Aronen, Jari Lipsanen, Minna Mannerkoski, Alexandra Hering, Sascha Zuber, Matthias Kliegel, Matti Laine and Juha Salmi in Journal of Attention Disorders

## Notes

1 The ADHD group included three participants with a secondary emotional diagnosis (F93/F98) and six participants with ongoing medication. To probe whether these secondary emotional diagnoses or ongoing medications could have affected the results, we examined the dependent variable mean values of these two small subgroups separately, and re-ran the group comparisons and the ROC analyses of main EPELI measures after excluding each subgroup at a time from the analysis. In both subgroups, the average performance on all five EPELI main measures fell very close (±0.3 standard deviations) to that of the ADHD group as whole. As regards the ANOVAs for the ADHD versus control group comparison, the results remained practically unchanged when one or the other subgroup was omitted from the analysis. Also for the ROC analyses, the areas under curve (AUCs) changed at most by two percent when leaving out one or the other subgroup. Together, this suggests that neither these comorbidities nor the additional medications confound the present results.
