# Supplementary material for: Quantifying ADHD Symptoms in Open-Ended Everyday Life Contexts With a New Virtual Reality Task
Source: J Atten Disord. 2021 Dec 5;26(11):1394–411. doi: 10.1177/10870547211044214 (PMC9304743; doi:10.1177/10870547211044214)
Supplement: sj-pdf-3-jad-10.1177_10870547211044214 – Supplemental material for Quantifying ADHD Symptoms in Open-Ended Everyday Life Contexts With a New Virtual Reality Task [file sj-pdf-3-jad-10.1177_10870547211044214.pdf]

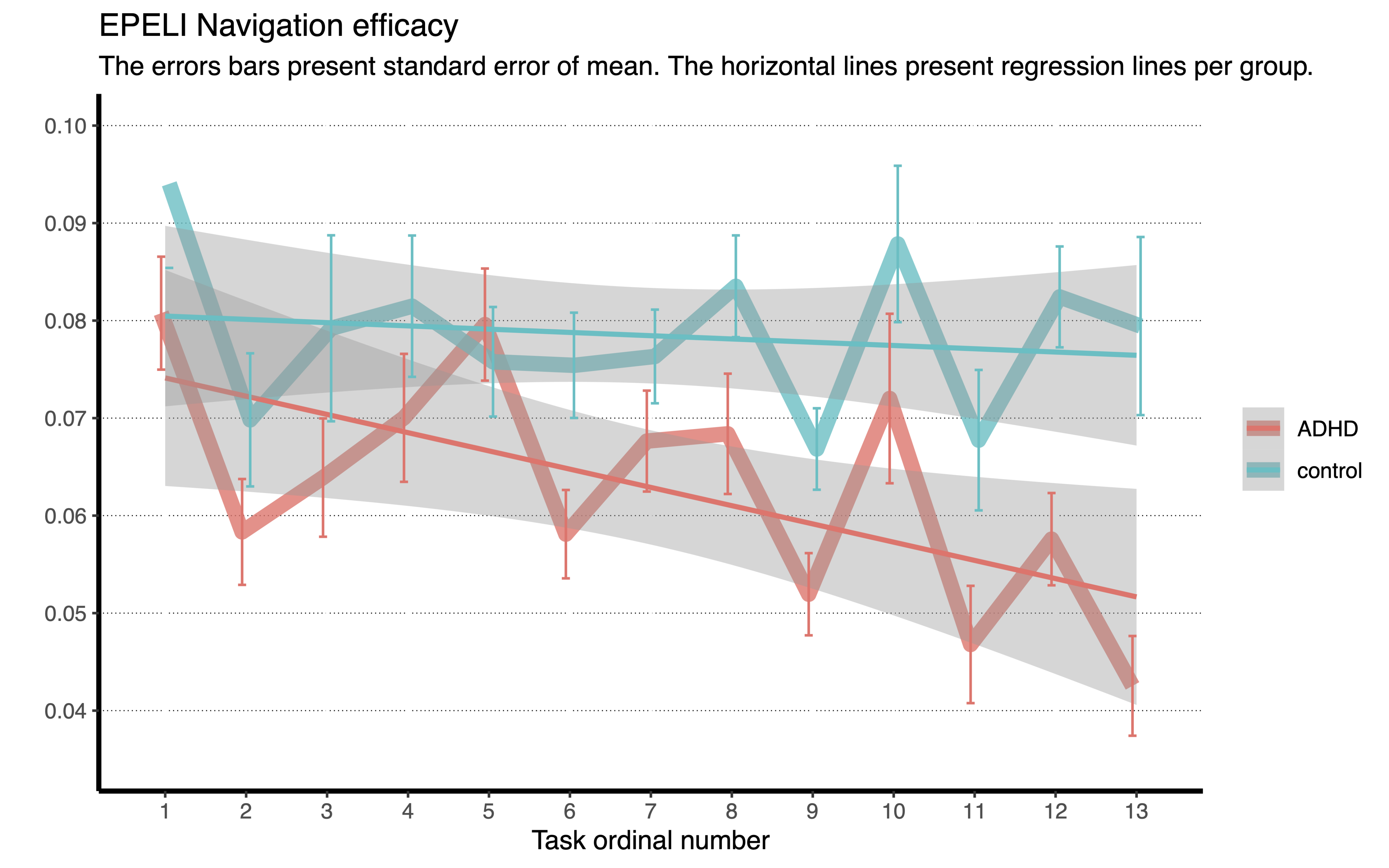

EPELI Navigation efficacy
The errors bars present standard error of mean. The horizontal lines present regression lines per group.
0.10
0.09
0.08
0.07
0.06
0.05
0.04
1
2
3
4
5
6
7
8
9
10
11
12
13
Task ordinal number
ADHD
control
