# Supplementary material for: Quantifying ADHD Symptoms in Open-Ended Everyday Life Contexts With a New Virtual Reality Task
Source: J Atten Disord. 2021 Dec 5;26(11):1394–411. doi: 10.1177/10870547211044214 (PMC9304743; doi:10.1177/10870547211044214)

EPELI Total actions

The errors bars present standard error of mean. The horizontal lines present regression lines per group.

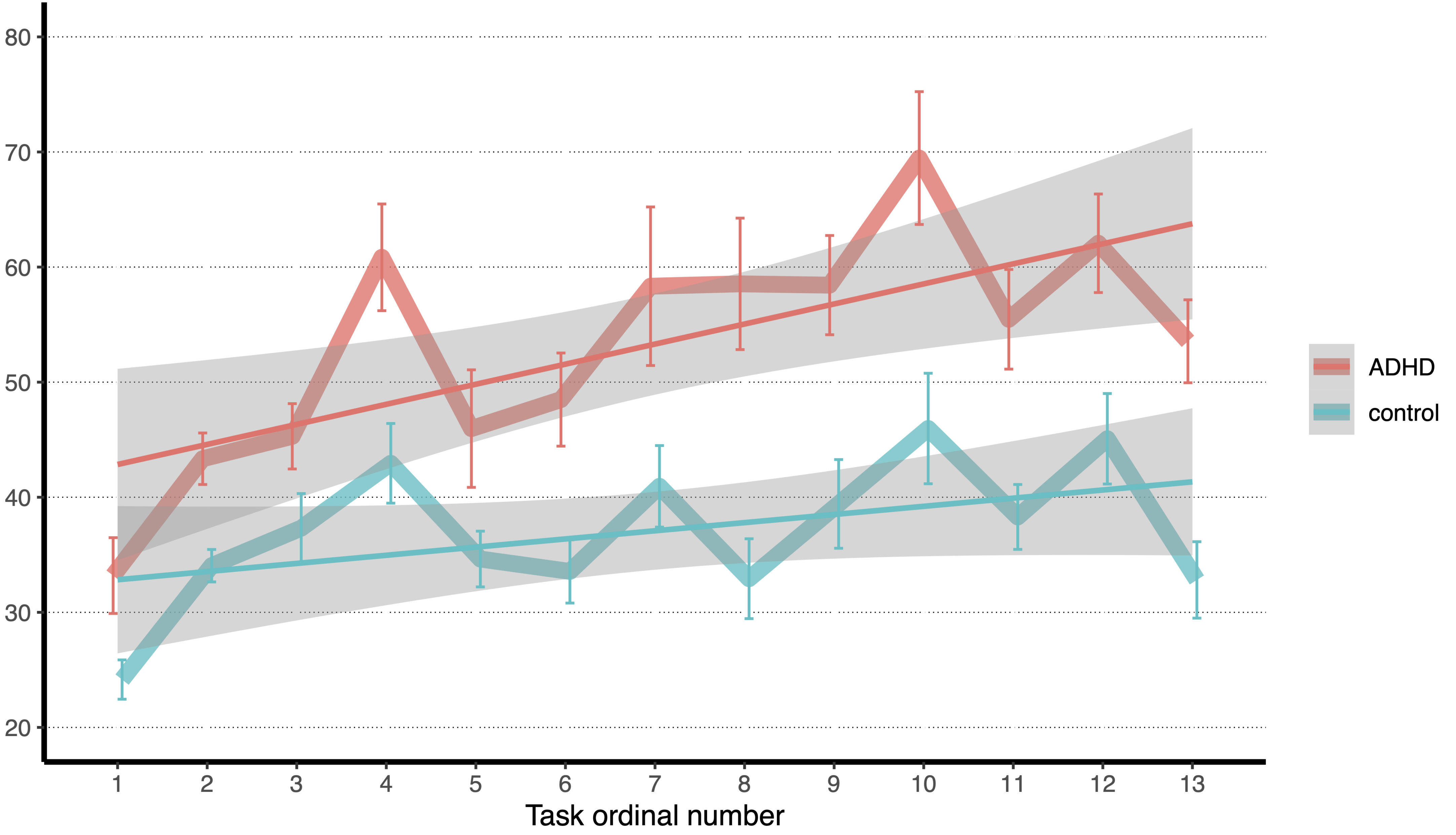

Supplement: sj-pdf-5-jad-10.1177_10870547211044214 – Supplemental material for Quantifying ADHD Symptoms in Open-Ended Everyday Life Contexts With a New Virtual Reality Task [file sj-pdf-5-jad-10.1177_10870547211044214.pdf]
