# Supplementary material for: Quantifying ADHD Symptoms in Open-Ended Everyday Life Contexts With a New Virtual Reality Task
Source: J Atten Disord. 2021 Dec 5;26(11):1394–411. doi: 10.1177/10870547211044214 (PMC9304743; doi:10.1177/10870547211044214)

Supplementary Methods. Parent and teacher ratings

Parents rated their child's ADHD symptoms using the ADHD Rating Scale-IV (ADHD-RS; DuPaul et al., 1998), which includes three subscales (inattention, hyperactivity, and impulsivity). Due to the limited sample size, high intercorrelations between the subscales, and high total number of variables in the study, we selected the total score (range 0–56) as the dependent variable for ADHD-RS. Everyday attention and executive function deficits were assessed using the Behavior Rating Inventory for Executive Functions (BRIEF; Gioia et al., 2000) also filled in by the parents. In BRIEF, the total score (Global Executive Index; range 72–216) was selected as the dependent variable. Teachers were also invited to fill out the ADHD-RS and BRIEF questionnaires, but because the answer rate was low (57 % for ADHD-RS and 38 % for BRIEF) probably partly due to difficult situation at the schools due to COVID-19 pandemic, the teacher ratings were not used in the main analyses. To query problems in the specific scenarios (e.g., preparing for school, cleaning the house) presented in EPELI, we designed a new parent questionnaire, the Executive Questionnaire of Everyday Life (EQELI; see Supplementary Table 4), from which the total score (range 0–100) was used as the dependent variable. Overall psychiatric symptoms were screened using the Child Behavior Checklist (CBCL; Achenbach, 1991).

Supplementary Methods. The conventional neuropsychological tasks

In the Similarities and Matrix reasoning subtests of the Finnish version of the Wechsler Intelligence Scale for Children (WISC-IV; Wechsler, 2003) participants solve reasoning problems that are presented as a verbal question or a visual matrix with one to-be-completed cell. These were conducted according to the test manual and used to assess general verbal and perceptual reasoning abilities.

In the present version of the Continuous Performance Task (CPT; Rosvold et al., 1956), participants were presented with a sequence of letters with fixed alternating intervals (1 s, 2 s, and 4 s). They were required to press the space bar for each letter, except for the letter X (probability of occurrence 10 %). For the main analyses, three dependent variables were included: omission errors, commission errors, and reaction time variability (standard deviation). For a multivariate classification analysis, we also included two other conventional CPT variables (mean reaction time and number of correct responses), to match the number of variables with a similar multivariate analysis conducted for the EPELI variables. There was a 30-trial practice session that was repeated twice if the participants achieved fewer than 20 correct responses. The actual task included 180 trials, and the task duration was approximately 7 min.

In the Simple Reaction Task (SRT), participants were instructed to press the space bar in response to a repeating visual stimulus (grey box with black sidelines) presented randomly at fixed alternating intervals (2 to 10 s) akin to the Psycho-Motor Vigilance task (PVT; e.g. Wilson et al., 2010). For the main analyses, mean reaction time was used as the dependent variable. The duration of the actual task was set at 4 min, resulting in approximately 36–42 trials depending on the reaction times in individual trials.

In the Cruiser, participants had to perform a time-based prospective memory task embedded in a driving game. We selected the Swiss Cruiser version which resembles the Dresden Cruiser (Kliegel et al., 2013; see also CyberCruiser in Kerns & Price, 2001). For the ongoing task, participants steered a car on a two-dimensional road of three parallel lanes by using the left and right arrow keys. They gained points by avoiding hitting other cars and overtaking them. The ongoing task was first practiced separately for 1 min, followed by another task (WISC-IV Digit Span). After the other task was completed, the time-based prospective memory task was presented: in addition to steering the car, participants were instructed to remember to refuel when the fuel gauge was on red. The fuel gauge was hidden, but by pushing the “c”-button on the keyboard, it appeared and remained on the screen for 3 s. Refueling was done by pressing the space bar during the last 10 s of every trial, which corresponded with the fuel gauge being red. Remembering to fill the tank resulted in additional points. If participants forgot to refuel, the tank was filled automatically. After the task, participants were to describe the task instructions to ensure that they remembered the prospective task retrospectively, which all participants

were able to do. In the main analysis, the number of successful refills was used as the dependent variable for time-based prospective memory performance. In total, the duration of the actual task was 4 min 15 s, consisting of four trials of 60 s plus 15 s overtime.

In the Frogs and Cherries Task (F&C), participants had to compare two halves of a 4 x 4 grid shown on a computer screen and answer which one had more pictures in it (see e.g., Dots & Triangles in Zuber et al., 2019). The task comprised two single task blocks (A and B) and a mixed-task block (C) performed in the same order (A, B, C) for all participants. In block A, participants had to decide whether there were more frogs on the left or right half of the grid by pushing the left or right arrow key. In block B, participants had to decide whether there were more cherries on the upper or lower half of the grid by pushing the up or down arrow key. Blocks A and B both included 10 practice trials, 10 more practice trials if the proportion of the correct answers was less than 60 %, and 40 experimental trials. In block C, participants had to shift between the two blocks: in the first four trials, the task was to decide whether there were more frogs on the left or right half of the grid, in the next four trials one had to decide whether there were more cherries on the upper or lower half of the grid, and so on. Block C included 17 practice trials, 17 more practice trials if the proportion of the correct answer was less than 60 %, and 81 experimental trials. The stimulus remained on the screen until a response was given, with a maximum response time of 4 s, and the next stimulus was presented immediately after each one was completed. The switching cost in RT was calculated by subtracting the mean reaction time on correct non-shift trials in block C (i.e., the trials where the task was the same as in the previous trial) from the mean reaction time on shift trials in block C (i.e., the trials where the task was different from the previous trial). For the main analyses, the switching cost RT was used as a dependent variable. The duration of the actual task was approximately 5 min, depending on the average response time of the participant.

In the Heidelberger Exekutivfunktionsdiagnostikum (HEXE) task, which is a modified and computerized variant of the Six Elements Task (Shallice & Burgess, 1991), participants needed to plan the delayed performance of difference subtasks and execute the plan independently later (Kliegel et al., 2006). The task comprised four phases. In the practice phase, four card decks with different tasks were presented in the lower part of the screen. Two blue decks contained math problems and two green decks contained picture tasks. One blue deck contained addition equations and the other subtraction equations presented in the middle of the screen with values between 10–99 (e.g., "27 + 17 = 44"), and the task was to state whether the equation was correct or not. One green deck contained pictures of objects (e.g., a knife or a spaceship) and the other pictures of animals/plants/places (e.g., an owl, a camel, or a volcano) presented in the middle of the screen, and the task was to respond whether the object/animal/plant/place in the picture was something commonly found in Finland. After a yes/no response, the next item of the same subtask was automatically presented. Participants

could switch between the decks by pressing four buttons (one for each deck), but they were only allowed to switch to a deck of different content (from picture to math deck or vice versa, but not from math to math or picture to picture; failure to follow this rule was recorded). The instructor showed an example with two different decks while explaining his/her actions. In the subsequent practice and planning phase, participants practiced the tasks in each deck. After this, the prospective task was explained: later, when the instructor would say “That was the second last task of today”, the participant would have to tell the instructor “Now we need to do that deck task”. Furthermore, while performing the task again with no repetition of the task instruction, participants would have to remember to do at least one item correctly in each of the four decks within the time limit of two minutes. Finally, participants were told that the bar at the upper part of the screen would start empty and be gradually filled in red. When the bar was be fully red, the time would be up. After the instructions, the children were asked to tell the instructor a step-by-step plan on how they could best succeed in completing the given task. During the retention phase, participants performed one of the other computerized tasks (CPT, SRT or F&C; depending on the counterbalanced task order for that given participant), after which they were asked to tell again their plan for managing “the deck task”. Then the participants performed another computerized task (CPT, SRT or F&C). After that, the instructor told participants that “That was the second last task of today” (the cue for starting the prospective memory task), and participants were supposed to remember to respond, “Now we need to do that deck task” (the initiation of the prospective memory task). If participants failed to respond appropriately, they were told that “Now we’ll do that deck task that you practiced before”. Then the task was started for them. During the execution phase, participants performed the HEXE task independently. For the main analysis, four different variables were used: 1) success in remembering to respond correctly to the cue, 2) success in answering correctly to one item in all four decks, 3) the number of correctly answered items in all four decks, and 4) the number of incorrectly answered items in all four decks. After the task, the participants were asked to repeat the instructions to ensure that they remembered the task correctly afterwards. Nine participants failed to do so and were excluded from the analysis.

The Clock Task was an event-based prospective memory task that was explained at the beginning of the test session and performed during and between the other tasks (for a similar task, see finger-snapping task in Kerns & Price, 2001). Participants were told that, while working on the tasks, they needed to monitor if the instructor put a wristwatch on the table next to the laptop used in the computerized tasks (the cue to the participant). Whenever the instructor did so, the participant needed to say “Boo”. This was practiced a couple of times before proceeding with the next task (WISC-IV Similarities). The first trial was during the WISC-IV Similarities task. If the child failed to respond to the cue at this first trial, he/she was asked “Did you have to remember something that was related to this wristwatch?”. If the child still failed to say

“Boo”, he/she was reminded about the task. There were three more trials in the Clock task, and they occurred between the other tasks. In the remaining three trials, the participant was not reminded of the task. After the last trial, the child was asked to repeat the task instructions. Two participants failed to remember this, and they were excluded from the analysis.

Supplementary Methods. EPELI floor plan

The apartment includes bathroom (upper left corner), living room with parents' bed, kitchen, child's room (right), and balcony that is not accessible but visible through windows (bottom).

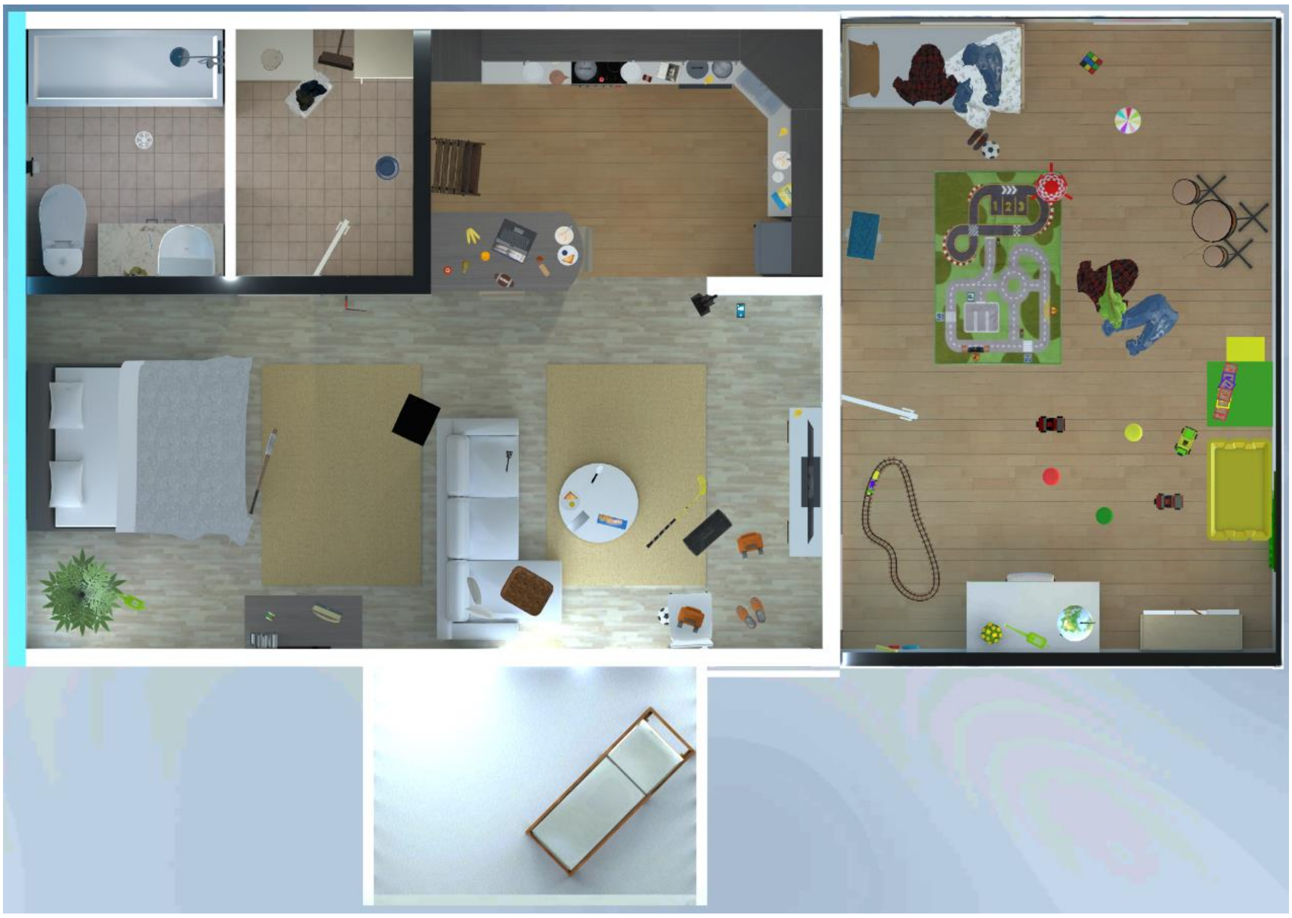

Supplement: sj-pdf-6-jad-10.1177_10870547211044214 – Supplemental material for Quantifying ADHD Symptoms in Open-Ended Everyday Life Contexts With a New Virtual Reality Task [file sj-pdf-6-jad-10.1177_10870547211044214.pdf]
