# Supplementary material for: Quantifying ADHD Symptoms in Open-Ended Everyday Life Contexts With a New Virtual Reality Task
Source: J Atten Disord. 2021 Dec 5;26(11):1394–411. doi: 10.1177/10870547211044214 (PMC9304743; doi:10.1177/10870547211044214)
Supplement: sj-pdf-7-jad-10.1177_10870547211044214 – Supplemental material for Quantifying ADHD Symptoms in Open-Ended Everyday Life Contexts With a New Virtual Reality Task [file sj-pdf-7-jad-10.1177_10870547211044214.pdf]

Supplementary Table 1.

Correlations of the EPELI measures and indices from which the main measures were selected (n=50, all controls before propensity matching). The selected main EPELI measures are written in bold, and the secondary set of prospective memory measures are written in italic.

	**Total score 1**	Total score 2	**Task efficacy 1**	Task efficacy 2	Task efficacy 3	**Navigation efficacy**	Head motion	**Controller motion**	Actions 1	Actions 2	**Actions 3**	*TBPM score*	*Clock checks*	*EBPM*
**Total score 1**	1.00													
Total score 2	.96	1.00												
**Task efficacy 1**	.27	.23	1.00											
Task efficacy 2	.47	.48	.84	1.00										
Task efficacy 3	.38	.37	.89	.96	1.00									
**Navigation efficacy**	.71	.66	.73	.83	.81	1.00								
Head motion	-.09	-.02	-.36	-.23	-.30	-.33	1.00							
**Controller motion**	-.08	-.01	-.46	-.27	-.36	-.36	.84	1.00						
Actions 1	-.18	-.14	-.84	-.81	-.82	-.62	.29	.38	1.00					
Actions 2	-.19	-.18	-.82	-.91	-.88	-.67	.24	.31	.89	1.00				
**Actions 3**	-.18	-.13	-.81	-.74	-.78	-.69	.49	.63	.89	.82	1.00			
TBPM score	.76	.74	.21	.43	.36	.44	-.11	-.10	-.21	-.21	-.18	1.00		
Clock checks	.19	.17	-.01	.04	.00	.10	.17	.08	.03	.09	-.01	.22	1.00	
EBPM	.43	.34	.23	.32	.31	.36	-.03	-.06	-.20	-.18	-.20	.11	-.04	1.00

Total score 1. The sum of correctly performed subtasks when event-based subtasks are included only when they are performed within 10 seconds from the triggering event, and time-based subtasks are included only when they are performed within +/- seconds from the target time.

Total score 1. The sum of correctly performed subtasks when all performed event-based and time-based subtasks are initially scored as 1. Furthermore, a time bonus is given if an event-based subtask is performed within 10 seconds from the triggering event, and if a time-based subtask is performed within +/- seconds from the target time. Time bonus is 1 if the deviation from the optimal time is 0, and it decreases linearly and is 0 if the deviation from the optimal time is 10.

Task efficacy 1. Calculated as "Relevant actions / (All actions on objects + empty clicks)". An action is defined here as a click or a swing (in the case of the drum kit) on an object. The number of relevant actions is defined by going through all the subtasks the participant has successfully completed, seeing how many actions each successfully completed subtask at least requires, and summing these together. Empty click is a click that is performed when the ray coming from the controller in the virtual environment is not pointing to any object with an interactive component, but for example to a wall. Theoretically, Task efficacy 1 can vary from 0.0 (no single subtask has been completed successfully but at least one action has been performed) to 1.0 (no single irrelevant action has been performed). Note that clicks related to moving around (i.e., clicking on the movement points marked on the floor) are not included.

Task efficacy 1. Calculated as "Total score 1 / Actions 1".

Task efficacy 2. Calculated as "Total score 1 / Actions 2".

Navigation efficacy. Calculated as "Total score 1 / Distance covered". Distance covered (centimeters) is the total walking distance in the game environment plus distance to each object that has been manipulated (it is possible to use all objects in the field of view from any distance).

Head motion. The amount of angular head movement in degrees during gameplay (NOT including the time dragon is giving the instructions).

Controller motion. The amount of angular controller movement in degrees (NOT including the time dragon is giving the instructions).

Actions 1. Calculated as "Number of clicks on objects + Number of times the drum kit in the game environment has been played by swinging the controller towards it".

Actions 2. In this measure, one action represents any number of consecutive clicks (or swings in the case of the drum kit) on the same target object. However, if any two consecutive clicks are separated by more than 2 seconds, they are treated as belonging to two separate actions.

Actions 3. Calculated as Actions 1, but including also moving clicks, empty clicks and all clicks performed during the time the dragon character is giving the instructions to each task block.

TBPM score. The sum of time-based subtasks that have been performed within +/- 10 seconds from the target time.

Clock checks. The number of times when the participant has made the clock visible by turning his/her hand upwards.

EBPM score. The sum of event-based subtasks that have been performed within + 10 seconds from the triggering event.

Supplementary Table 2.

The group means and standard deviations of the five EPELI measures and the conventional neuropsychological tasks

	ADHD group		TD group	
Variable	*Mean*	*SD*	*Mean*	*SD*
EPELI Total score	45.1	7.6	50.8	6.5
EPELI Task efficacy	0.183	0.087	0.344	0.142
EPELI Navigation efficacy	0.055	0.014	0.0704	0.017
EPELI Controller motion	80001.1	22768.0	61908.7	14912.6
EPELI Total actions	693.0	229.8	482.1	180.9
Digit span	12.2	2.2	13.8	2.8
Repetition task	27.1	7.8	29.3	10.5
CPT omissions	11.1	11.2	4.5	4.1
CPT commissions	13.2	3.6	10.7	3.6
CPT RT variability	276.1	153.5	122.2	35.0
SRT mean RT	598.4	173.6	503.6	91.0
F&C switching cost	264.0	264.2	104.0	133.5
Cruiser PM accuracy	0.7	0.3	1.0	0.1
Cruiser monitoring	19.2	9.3	23.7	7.8
Cruiser number of crashes	4.9	4.0	3.1	1.9
Clock task PM accuracy	3.2	1.0	3.3	0.9
HEXE correct task responses	31.3	20.8	25.4	13.9
HEXE ongoing errors	4.2	2.9	2.7	2.7
HEXE self-initiated PM task[1]	22 / 3		28 / 7	
HEXE switching PM task[1]	22 / 3		31 / 3	

PM, prospective memory, RT, reaction time. [1] the number of participants who correctly performed the PM task / the number of participants who failed to perform the PM task.

Supplementary Table 3.

The correlations between main EPELI measures and conventional neuropsychological measures not yielding group differences

	WISC IV Similiarities	WISC IV Matrix reasoning	Repetition task	Cruiser monitoring	Clock task PM accuracy	HEXE correct task responses	HEXE self-initated PM task	HEXE switching PM task
Total score	.286 *	.306 *	.485 ***	.290 *	.219	.038	.008	.039
Task efficacy	.286 *	.022	.340 *	.132	.089	-.296	.129	.028
Navigation efficacy	.311 *	.215	.505 ***	.116	.170	-.165	-.005	-.036
Controller motion	-.120	-.067	-.333 *	-.025	-.081	.345 *	-.036	-.073
Total actions	-.268	-.097	-.299 *	-.055	.054	.339 *	-.083	-.033

* $p \leqq .05$. ** $p \leqq .01$. *** $p \leqq .001$. FDR correction.

Supplementary Table 4.
The English translation of the Executive Questionnaire of Everyday Life (EQELI) developed for the present study. The total score (0–100) is the sum of the sub questions from the questions 1–4.

Child's name ___________________
Filler of the questionnaire (circle): mother / father / other, who ________

Please read each statement and circle the answer that applies the best (0 = never, 1 = rarely, 1 = sometimes, 2 = often, 3 = very often).

	never	rarely	sometimes	often	very often
1. Difficulties in self-directed behavior and taking initiative in the following situations:					
morning activities, going and returning from school	0	1	2	3	4
going and returning from outdoor activities and hobbies	0	1	2	3	4
housework (for example, cleaning, cooking)	0	1	2	3	4
evening activities, going to sleep	0	1	2	3	4
other situations	0	1	2	3	4
2. Difficulties in remembering to do all the required tasks in the following situations:					
morning activities, going and returning from school	0	1	2	3	4
going and returning from outdoor activities and hobbies	0	1	2	3	4
housework (for example, cleaning, cooking)	0	1	2	3	4
evening activities, going to sleep	0	1	2	3	4
other situations	0	1	2	3	4
3. Difficulties in carrying out preparations in appropriate order in the following situations:					
morning activities, going and returning from school	0	1	2	3	4
going and returning from outdoor activities and hobbies	0	1	2	3	4
housework (for example, cleaning, cooking)	0	1	2	3	4
evening activities, going to sleep	0	1	2	3	4
other situations	0	1	2	3	4
4. Difficulties in sticking to the schedule in the following situations:					
morning activities, going and returning from school	0	1	2	3	4
going and returning from outdoor activities and hobbies	0	1	2	3	4
housework (for example, cleaning, cooking)	0	1	2	3	4
evening activities, going to sleep	0	1	2	3	4
other situations	0	1	2	3	4
5. Difficulties in ignoring distractive stimuli (noises, other people) in the following situations:					
morning activities, going and returning from school	0	1	2	3	4
going and returning from outdoor activities and hobbies	0	1	2	3	4
housework (for example, cleaning, cooking)	0	1	2	3	4
evening activities, going to sleep	0	1	2	3	4
other situations	0	1	2	3	4
6. Difficulties in completing pleasant tasks, if they contain multiple stages	0	1	2	3	4
7. Difficulties in coping in pleasant hobbies or free time tasks	0	1	2	3	4

We are developing this questionnaire. If you wish, you can write feedback of the questionnaire below:
____
____

Supplementary Table 5.
The English translation of the Gaming background questionnaire

1) Have you played with VR-goggles before? (yes/no)
   If yes, do you play with them regularly? (yes/no)
   If yes, how many days per week?

2) Approximately how long do you play per session (in minutes)?

3) How many years have you been playing regularly?

4) Have you played computer, console, or mobile games? (yes/no)
   If yes, do you play them regularly? (yes/no)
   If yes, how many days per week?

5) Approximately how long do you play per session (in minutes)?

6) How many years have you been playing regularly?

7) (Ask only if child has answered that he/she plays games)
   What games do you play?

Supplementary Table 6.
Child Simulator Sickness Questionnaire

All questions were answered with three-point scale (No = 0; A little = 1; A lot = 2). These are the original questions (Hoeft et al., 2003). A Finnish translation was employed in the present study.

	ADHD group			TD group		
Question	*Median*	*Mean*	*SD*	*Median*	*Mean*	*SD*
1. Do you feel sick?	0	0.11	0.39	0	0.11	0.31
2. Does your head hurt?	0	0.18	0.46	0	0.26	0.45
3. Do your eyes hurt?	0	0.16	0.37	0	0.21	0.47
4. Do you have an upset stomach?	0	0.03	0.16	0	0.08	0.27
5. Are you dizzy with your eyes open?	0	0.08	0.27	0	0.13	0.34
6. Are you dizzy with your eyes closed?	0	0.08	0.27	0	0.05	0.23
7. Are you burping at all?	0	0.00	0.00	0	0.05	0.23

Supplementary Table 7.
The Presence Questionnaire

This questionnaire is based on Presence Questionnaire 3.0 (see Witmer, Jerome, and Singer 2005 for revised factor structure). The first nine questions appear on the original questionnaire as such or slightly differently. If the question has been altered to be more suitable for children or the shortened version, the original form is presented in parentheses. A Finnish translation was employed in the present study.

The following instruction is read by the experimenter: "I'm going to ask you some questions regarding the game. You can answer by choosing the best alternative from the scale of 1–7 you can see on the screen. The margin on the left means "no" and the margin on the right means "completely/very much", the rest of the alternatives are between those."

	ADHD group			TD group		
Question	*Median*	*Mean*	*SD*	*Median*	*Mean*	*SD*
1. How natural did your interactions with the environment seem?	5	4.79	1.85	5	4.89	1.09
2. How much did the environment involve you? (How much did the visual aspects of the environment involve you? & How much did the auditory aspects of the environment involve you?)	5	5.05	1.61	6	5.18	1.63
3. How natural was the mechanism which controlled movement through the environment?	4	3.87	1.73	4.5	4.18	1.80
4. How much did your experiences in the virtual environment seem consistent with your real-world experiences?	5	4.92	1.53	5.5	5.29	1.33
5. How much did the visual display quality interfere or distract you from performing assigned tasks or required activities?	1	2.34	1.83	2	2.42	1.43
6. How much did the control devices interfere with the performance of assigned tasks or with other activities?	1	2.03	1.73	1	1.87	1.30
7. How well could you concentrate on the assigned tasks or required activities? (How well could you concentrate on the assigned tasks or required activities rather than on the mechanisms used to perform those tasks or activities?)	5	5.08	0.66	6	5.58	0.63
8. How well could you hear sounds? (How well could you identify sounds? & How well could you localize sounds?)	6	6.68	0.66	7	6.76	0.63
9. Were there moments during the virtual environment experience when you felt completely focused on the task or environment?	5	4.16	2.51	5	4.34	2.02
Three additional questions that were not in the original Presence Questionnaire 3.0:						
10. How enthusiastic did you feel about the tasks?	6	5.00	1.95	6	5.45	1.59
11. How interesting did the tasks seem to you?	5.5	4.82	2.17	6	5.32	1.54
12. How much effort did you put into your performance?	6	5.87	1.23	6	6.21	0.96

Supplementary Table 8.
Object recognition task

The following instruction is read by the experimenter: “Next, I’m going to show you pictures of the game and ask you to name some objects. Each object from the list was shown to the child with the question “What is this?” If the child could not answer in 10 seconds, the experimenter asked, “What can you do with it?” With every object, the experimenter also asked: “How fun was it? Answer with the first thing that comes up to your mind and choose the best emoji to describe it.”

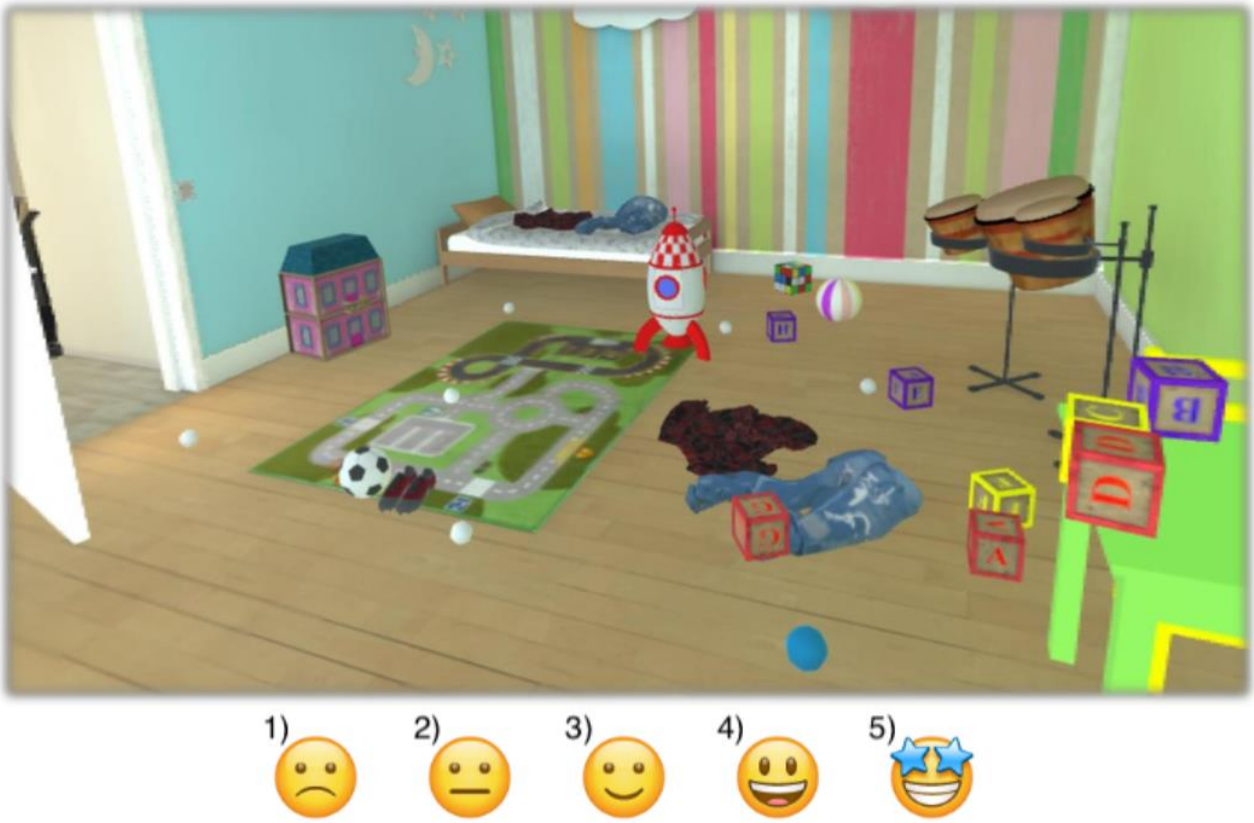

Picture 1. *The target objects include football, soccer shoes, beach ball, drums, light switch.*

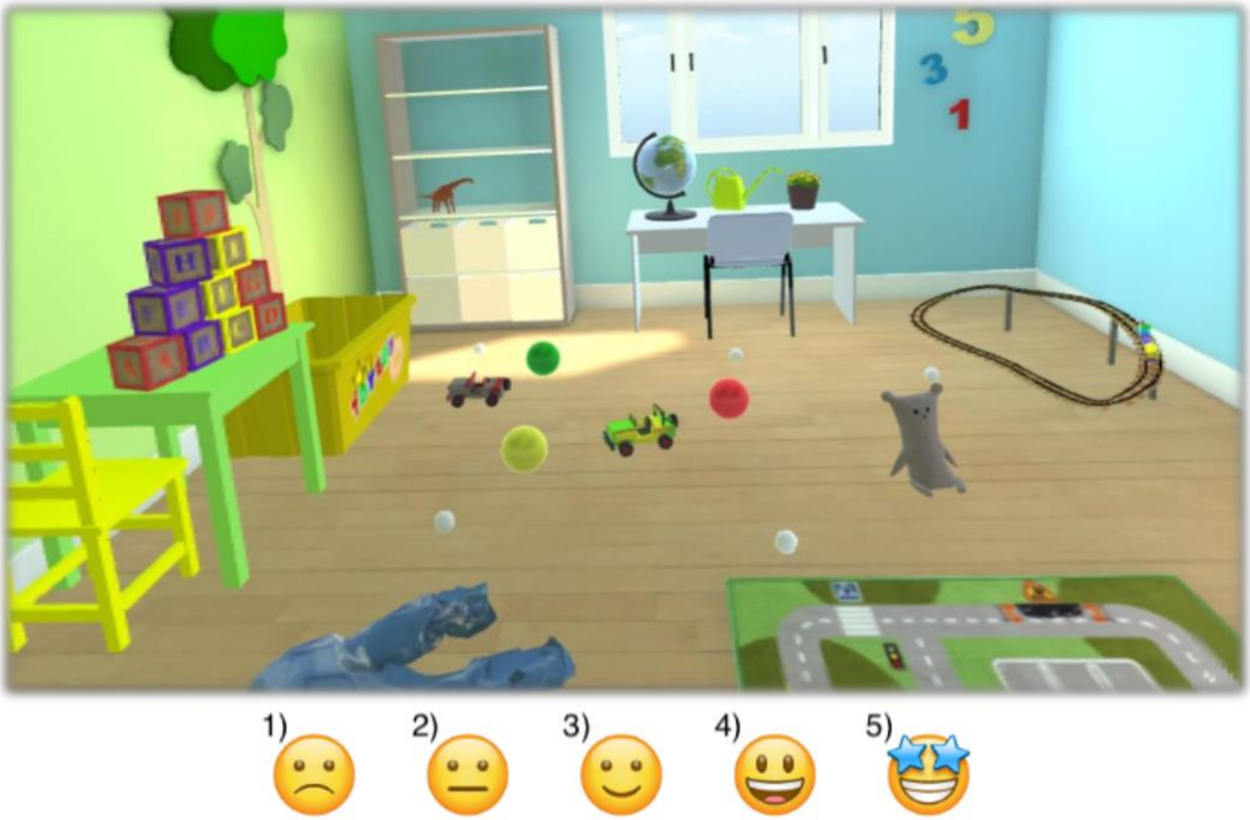

Picture 2. *Target objects include block tower, globe, toy train / railway track, teddy bear, toy car.*

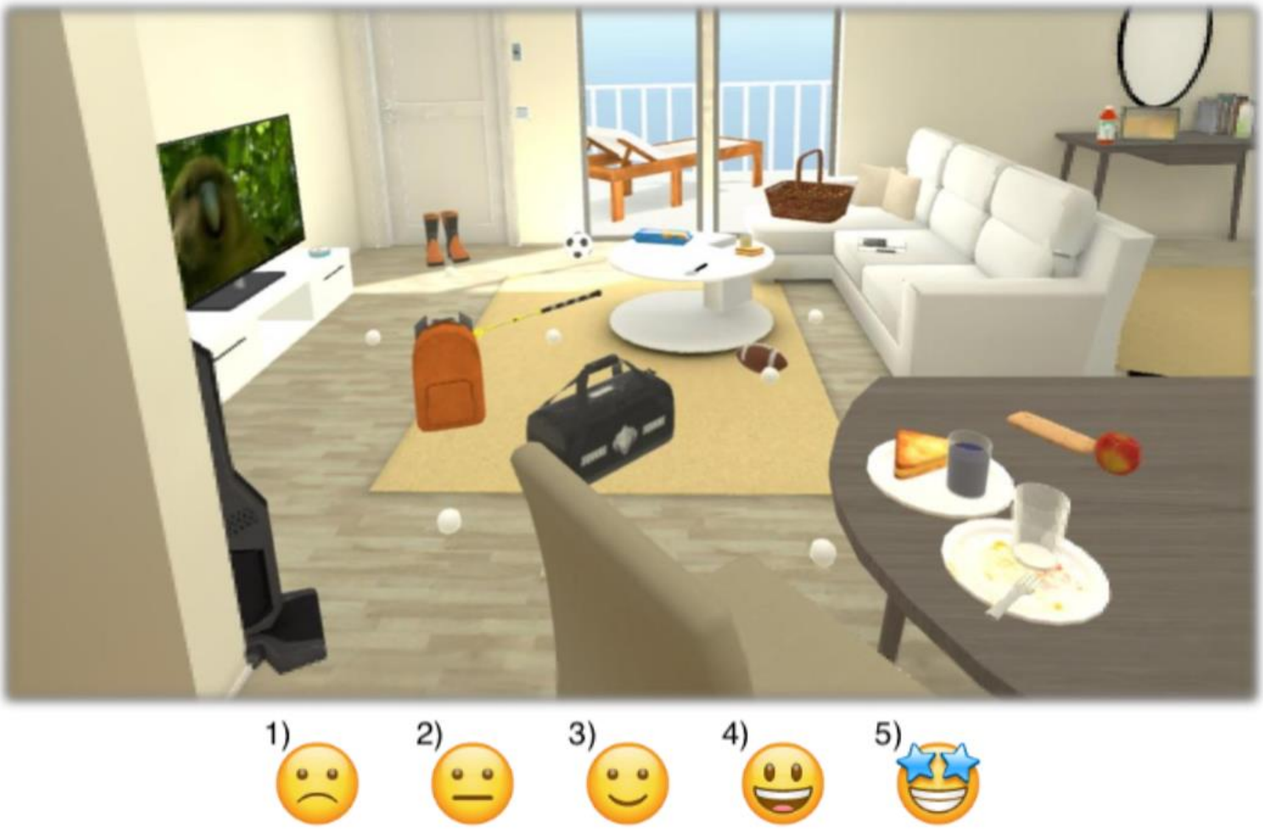

Picture 3. *The target objects include TV, schoolbag, radio, apple.*

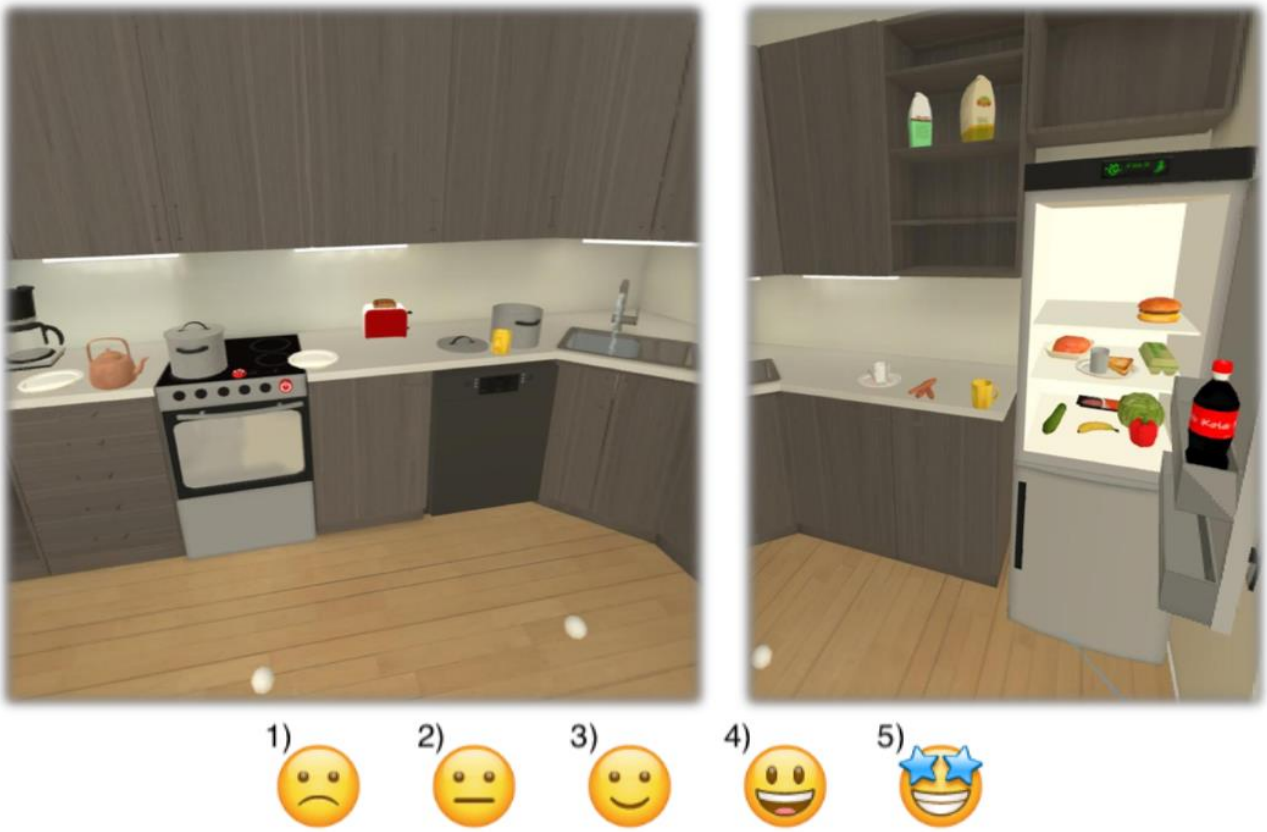

Pictures 4 and 5. *Target objects include carrots, Coca Cola bottle, dishwasher, oven / stove.*

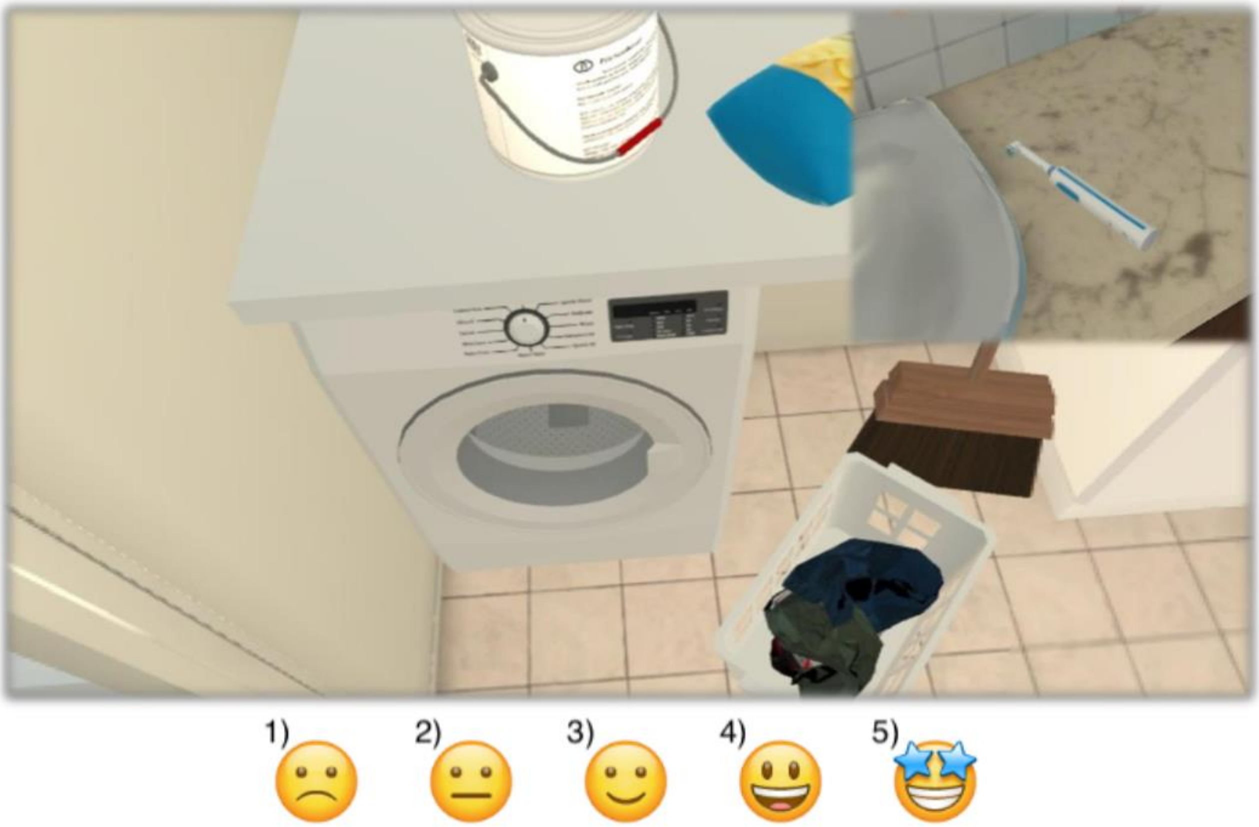

Pictures 6 and 7. *Target objects include laundry basket and toothbrush.*
